# Urinary Metabolic Profiling via LC-MS/MS Reveals Impact of Bovine Lactoferrin on Bone Formation in Growing SD Rats

**DOI:** 10.3390/nu12041116

**Published:** 2020-04-17

**Authors:** Yan Xu, Tianyu Zhao, Haowei Ren, Yindan Xie, Jingjing An, Jiaqi Shang, Dina Tabys, Ning Liu

**Affiliations:** 1 Key Laboratory of Dairy Science, Ministry of Education, Northeast Agricultural University, Harbin 150030, China; xuyan1991521@163.com (Y.X.); 13091456816@163.com (T.Z.); renhw800903@126.com (H.R.); xieyindan99@163.com (Y.X.); anjingloveme@163.com (J.A.); ashang10@163.com (J.S.); tabysdina@gmail.com (D.T.); 2 College of Food Science, Northeast Agricultural University, Harbin 150030, China

**Keywords:** lactoferrin, metabolomics, bone formation, osteogenesis, LC-MS/MS

## Abstract

Lactoferrin (LF) exerts a promoting bone health function. The effects of LF on bone formation at the metabolic level have been less explored. Urinary metabolic profiling of growing Sprague-Dawley (SD) rats LF-supplemented (1000 mg/kg bw) for four weeks were explored by Liquid chromatography–tandem mass spectrometry (LC-MS/MS). The serum markers of bone formation and bone resorption, the bone mass, and the osteogenesis markers of femur were measured by an enzyme-linked immunosorbent assay, micro-computerized tomography, and immunohistochemistry, respectively. Compared with the control, LF supplementation improved bone formation (*p* < 0.05), reduced bone resorption (*p* < 0.05), enhanced femoral bone mineral density and microarchitecture (*p* < 0.05), and upregulated osteocalcin, osterix, and Runx-2 expression (*p* < 0.05) of femur. LF upregulated 69 urinary metabolites. KEGG and pathway enrichment analyses of those urinary metabolites, and the Person’s correlation analyses among those urinary metabolites and bone status revealed that LF impacted on bone formation via regulatory comprehensive pathways including taurine and hypotaurine metabolism, arginine and proline metabolism, cyanoamino acid metabolism, nitrogen metabolism, nicotinate and nicotinamide metabolism, and fatty acid biosynthesis. The present study indicated the metabolomics is a useful and practical tool to elucidate the mechanisms by which LF augments bone mass formation in growing animals.

## 1. Introduction

Bone has intrinsic capacities for regeneration, remodeling, and self-repair. These processes ensure that bone tissue and its metabolism develop normally and maintain healthy mechanical skeletal function. Bone mass increases during childhood and adolescence until peak bone mass is reached, and increased peak bone mass reduces the risk of osteoporosis later in life [1,2]. Therefore, improving bone growth by diet and nutrition in the early life, especially in infancy, childhood, and adolescence, may substantially influence bone health later in life [3,4,5].

Lactoferrin (LF) is a multifunctional iron-binding glycoprotein (MW 80 kDa) in the transferrin family, which is present in milk, saliva, and other exocrine secretions [6,7]. LF enhances proliferation and differentiation of osteoblasts (bone-forming cell) [8,9] and mesenchymal stem cells (osteogenic bone progenitors) [10,11] and inhibits osteoclast (bone-resorbing cell) [12] in vitro. LF significantly promotes bone regenerative capacity [13,14], improves bone mass and microarchitecture [15], and reduces bone resorption [16,17] in vivo. These findings suggest that LF, as a nutrient, is a potent anabolic bone formation and growth factor, and plays a role in improving bone health. Some previous studies have attempted to explore the mechanisms by which LF promotes osteogenic differentiation in vitro [9,18,19,20]. Osteogenic differentiation is precisely regulated and orchestrated by the mechanical and molecular signals from the extracellular environment; however, the mechanism of orally LF promoting bone formation in vivo is unclear and warrants study. Moreover, dietary nutrition components play a particularly important role in metabolism, but there are virtually no reports about the effect of LF on bone formation at the metabolic level. Therefore, it is of great significance to explore the mechanism of LF promoting bone formation from metabolic level.

Metabolomics identifies small-molecule metabolites in biospecimens, which are downstream of genomic and proteomic activity that characterize biological systems and represent overall physiological status. It has attracted interest in fields such as diagnostics [21], pathology [22], toxicology [23], and nutrition [24]. The effects of dietary biomarker identification [25], diet-related disease diagnostics [26,27,28], and nutritional interventions [29,30] have been studied using a metabolomic approach. The application of metabolomics in nutrition research helps to elucidate the effects of nutrients on metabolic regulation and profiling, and to integrate metabolic endpoints in response to nutrient absorption, metabolism, and disposition. This systems approach to studying nutrition reveals the mechanisms of biological functions, which enables disease prevention and the maintenance of health via appropriate nutrition. We therefore chose a metabolomic approach to explore the mechanisms by which LF promotes bone health, by studying the impact of supplementary LF on bone formation in growing SD rats. 

The aims of this study were to identify global changes in the urinary metabolic profiles of growing SD rats LF-supplemented (1000 mg/kg bw) for four weeks using non-targeted metabolomic platform, and to explore the metabolic pathways affected by LF to elucidate the mechanisms by which LF promotes bone growth and health in growing SD rats. To verify the effects of LF on bone formation, related serum bone turnover markers were evaluated by ELISA, bone mass was evaluated by μ-CT, and osteogenic differentiation markers of osteoblast were evaluated by immunohistochemistry (IHC). The present study lays the foundation for subsequent research on the modes of action of LF in bone growth and health promotion, and the mechanisms by which LF enhances bone formation in growing period. These results corroborate the use of nutritional LF as a functional food for promoting bone formation during early life, and in bone tissue engineering for the prevention of senile osteoporosis and the maintenance of bone health.

## 2. Materials and Methods

### 2.1. Chemicals and Reagents

Bovine lactoferrin (bLF) (purity ≥ 96.3%, iron saturation 15%) was obtained from Westland Cooperative Dairy Co. Ltd. (Hokitika, New Zealand). High-performance liquid chromatography (HPLC)-grade acetonitrile, methanol, ammonium acetate, ammonium hydroxide, and formic acid were purchased from CNW Technologies GmbH (Düsseldorf, Germany). 2-chloro-*L*-phenylalanine (purity ≥ 98%) was purchased from Shanghai Hengbai Biotechnology Co. Ltd. (Shanghai, China). The other chemicals used in this study were of analytical grade.

### 2.2. Animal Experimental Design

Twenty male Sprague-Dawley (SD) rats aged 4 weeks were purchased from Vital River Laboratory Animal Technology Co. Ltd. (Beijing, China) (license # SCXK (JING) 2016–0006). The rats were housed in an animal room maintained at 24 ± 2 °C, 50 ± 10% relative humidity (RH), and an alternating 12 h/12 h light/dark cycle.

After acclimation for 7 days, the rats were fed standard rat chow (AIN93G). Rats were randomly assigned to the control (*n* = 10) group or the lactoferrin (*n* = 10) group. Rats were treated daily with either vehicle (normal saline; the control group) or lactoferrin (1000 mg/kg bw; the lactoferrin group) by oral gavage for 4 weeks. The dose of LF was selected using a reference maximum safe dose toxicity test [31].

On Week 4, after LF supplementation, rats were placed in metabolic cages to collect 24-h urine samples for the metabolomic study. All urine samples were separated by centrifugation at 645× *g* for 10 min at 4 °C. The supernatants were stored at −80 °C. All rats were fasted for 12 h and anesthetized by intraperitoneal sodium pentobarbital injection (40 mg/kg bw). Blood samples were drawn from the abdominal aorta and the serum were separated by centrifugation at 645× *g* for 15 min at 4 °C. After the animals were euthanized, the left femur of each rat was excised, freed from the muscle and connective tissue, fixed in 4% (*v*/*v*) paraformaldehyde, and stored in 70% (*v*/*v*) ethanol until μ-CT analysis. The right femurs were prepared for hematoxylin-eosin (H&E) staining and immunohistochemical (IHC) analysis.

Animal experiments were conducted with the approval of the Animal Ethics Committee of Northeast Agricultural University (Permit Number: 20181121-02), Harbin, China. Animal care and experimentation were performed in accordance with the Guidelines for the Care and Use of Laboratory Animals at Northeast Agricultural University, Harbin, China.

### 2.3. Measurement for Serum Markers of Both Bone Formation and Resorption by ELISA

Procollagen type I *N*-terminal propeptide (PINP), carboxyl-terminal telopeptide of type 1 collagen (CTX-1), bone alkaline phosphatase (BALP), and tartrate-resistant acid phosphatase (TRACP) in the rat serum were measured by enzyme linked immune sorbent assay (ELISA) kits according to the manufacturer’s instructions (Beijing Chenglin Biological Engineering Institute Co. Ltd., Beijing, China) and measured by microplate reader scanning (Labsystem Multiskan MS Primary EIA Version 1.5-0, Helsinki, Finland)) at λ = 450 nm.

### 2.4. Microcomputed Tomographic (μ-CT) Measures for Bone Analysis

The prepared femurs (*n* = 10/per group) were measured by microcomputed tomography (μ-CT) scan (ZKKS-MCT-SHARP, Guangzhou Zhongke Kaisheng Medical Technology Co. Ltd., Guangzhou, China). Images were acquired with 3D Med v. 2.0 (Guangzhou Zhongke Kaisheng Medical Technology Co. Ltd., Guangzhou, China). The method used was described in detail by Jing et al. [32]. The scans were obtained at 60 kV X-ray voltage, a focused spot diameter of 5 μm, a beam angle of 45°, and a resolution of 15 × 15 × 15 μm^3^. Section images were obtained continuously at 360°. The region of interests (ROI) were selected at 1 and 6 mm below the femoral bone growth plate using a layer thickness of 5 mm. Bone mineral density (BMD, g cm^−2^), percentage of bone volume (BV/TV; %) relative to the total measured area, trabecular thickness (Tb.Th, mm), trabecular number (Tb.N, mm^−1^), trabecular spacing (Tb.Sp, mm), and cortical thickness (Ct.Th, mm) were obtained from the ROIs. Morphometry of 3D images was obtained. 

### 2.5. Hematoxylin and Eosin (H&E) for Bone Staining

H&E staining was applied for observation of bone histological cells according to previously reported [33]. Briefly, the fixed bone samples were rinsed in various gradients of glycerin/PBS [1:5%, 2:10%, 3:15% (*v*/*v*)] and decalcified in 10% (*w*/*v*) EDTA for 4 weeks. The samples were washed for 24 h, dehydrated with an ethanol gradient (50%, 70%, 80%, and 100%), cleared with xylene, embedded in paraffin, and cut into 6-μm sections for use in subsequent histological and IHC analyses. The H&E-stained slides were imaged with a Moticam 3000 microscope photomicrography system (Motic, Richmond, BC, Canada).

### 2.6. Immunohistochemical (IHC) Analysis of Osteocalcin, Osterix, and Runx-2 Expression

To identify the osteogenesis markers of femur in SD LF-supplemented rats, the expression levels of osteocalcin, osterix, and Runx-2 in femur were measured by IHC according to a previously reported method with slight modification [34]. Briefly, the paraffin-embedded slices around the distal femurs were quenched in 3% (*v*/*v*) H_2_O_2_ for 10 min in methanol before serum blocking. After overnight incubation at 4 °C with the primary antibodies anti-Runx 2, anti-osteocalcin, or anti-osterix (Wuhan Bosider Biotechnology Co. Ltd., Wuhan, China), the sections were washed with phosphate-buffered saline (PBS) before incubation at 37 °C for 30 min with the secondary antibody PV-6001 goat anti-rabbit IgG-HRP multimer (Zhongshan Bio-tech Co. Ltd., Beijing, China). The sections were then incubated for 5–10 min with diaminobenzidine, rinsed with distilled water, counterstained with hematoxylin for 1 min, and viewed under a Moticam 3000 microscope photomicrography system (Motic, Richmond, BC, Canada). Quantification of the brown staining for target protein was evaluated by the Integral Optical Density (IOD) based on Image Pro Plus (IPP) v. 6.0 software (Media Cybernetics Inc., Rockville, MD, USA) from five random fields per slide (×200 magnification).

### 2.7. Urinary Metabolite Extraction

Samples were pretreated according to a previously reported method [35]. One hundred microliters urine sample and 400 μL extraction solvent (V_methanol_:V_acetonitrile_ = 1:1; 2 μg/mL 2-chloro-*L*-phenylalanine internal standard) were mixed and vortexed for 30 s, ultrasonicated for 10 min, and centrifuged at 12,000× *g* for 15 min. The supernatants (425 μL) were then separated and dried. They were reconstituted and the supernatants (60 μL) and 10 μL from each sample were pooled as QC samples and used in the subsequent ultra-high performance liquid chromatography/quadrupole time-of-flight mass spectrometry (UHPLC-QTOF-MS) analysis.

### 2.8. Urine Metabolic Profiling Analysis by UPLC-MS/MS

Urinary metabolic profiling analysis was performed on an Agilent 1290 UHPLC system (Agilent Technologies, Santa Clara, CA, USA) with a UPLC BEH amide column (1.7 μm; 2.1 mm × 100 mm; Waters Corp., Milford, MA, USA) coupled to a TripleTOF 6600 (Q-TOF; AB Sciex LLC, Redwood City, CA, USA) in simultaneous positive and negative ion modes. Mobile phase A consisted of 25 mM NH_4_Ac and 25 mM NH_4_OH in ultrapure water (pH 9.75). Mobile phase B consisted of acetonitrile. The UPLC conditions are shown in Table S1. The sample injection volume was 1 μL. MS data were collected with Analyst TF v. 1.7 (AB Sciex LLC, Redwood City, CA, USA) on an information-dependent basis (IDA) from MS/MS spectra.

The ESI source operating parameters were as follows: ion spray voltage floating = 5000 V or −4000 V in positive or negative modes, respectively; collision energy = of 30 V; ion source gas 1 at 414 kPa; ion source gas 2 at 414 kPa; curtain gas at 241 kPa; and source temperature = 600 °C. MS/MS spectrum acquisition depended on preselected criteria. Full MS scan data were obtained for a mass-to-charge ratio range of 50–1200 *m/z*.

### 2.9. Urine Metabolomic Data Processing and Annotation

Raw LC-MS data (wiff.scan) files were collected with Analyst TF v. 1.7 (AB Sciex LLC, Redwood City, CA, USA), converted to mzXML files with ProteoWizard MSConvert, and processed with the XCMS package in R v. 3.2 [36,37]. The data matrix included retention times (RT), mass-to-charge ratios (*m/z*), and peak intensities. The CAMERA package in R was used for peak annotation after XCMS data processing and metabolite identification using the in-house MS2 (Shanghai Biotree Biotechnology, Shanghai, China), online HMDB (http://www.hmdb.ca/), and KEGG (www.genome.jp/kegg/) databases [38].

SIMCA v. 14.1 (Sartorius Stedim Data Analytics AB, Umea, Sweden) was used for principal components analysis (PCA) and orthogonal projections to latent structures–discriminant analysis (OPLS-DA) for multivariates. Sevenfold cross-validation and the permutation test estimated and validated model robustness and predictive ability. The variable influence on projection (VIP) values was obtained from the OPLS-DA model. Two-tailed unpaired Student’s *t*-test was used to analyze the data. Differential metabolites were selected when VIP values were > 1.0, and *p*-values < 0.05. KEGG (http://www.genome.jp/kegg/) and MetaboAnalyst (http://www.metaboanalyst.ca/) were searched for metabolite pathways [39].

### 2.10. Statistical Analyses

The experiments were performed in three independent replicates (*n* = 10 rats per group). SPSS v. 22.0 (IBM Corp., Armonk, NY, USA) was used to test the data for normal distribution by the Shapiro-Wilk normality test for ELISA, micro-CT, and IHC. The data were then analyzed by an unpaired, two-tailed Student’s *t*-test and reported as means ± standard error of the mean (SEM). Each data point represents one rat. Differences between treatment means were considered significant at *p* < 0.05 and extremely significant at *p* < 0.01. Correlation between the bone status marker and urine metabolites was measured by Pearson correlation using the software R version 3.6.1 and the correlation matrix plot was created using Hmisc, and ggcorrplot packages.

## 3. Results

### 3.1. LF Improved Bone Growth in Growing SD Rat

**Figure 1:**
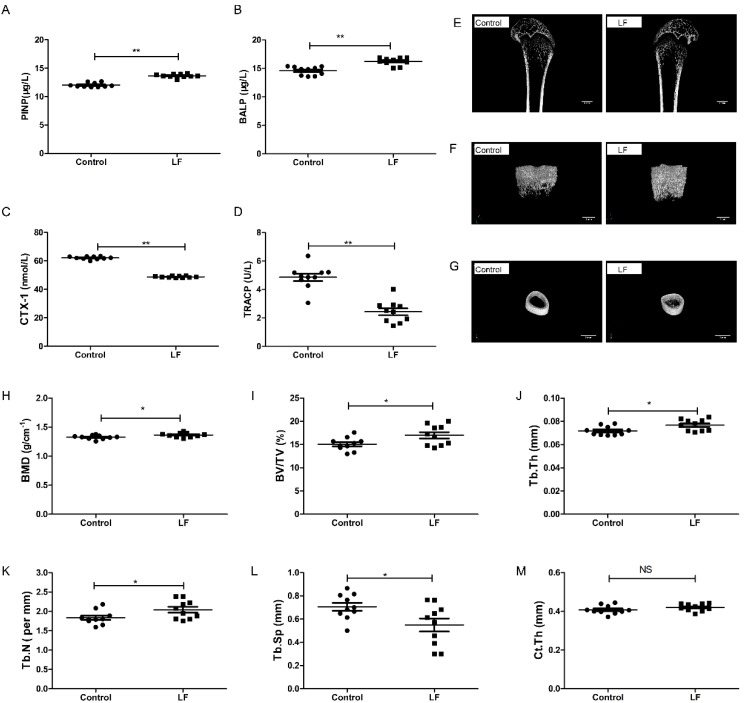
LF supplementation enhanced bone formation, reduced bone resorption, and induced bone mass microarchitecture. Growing SD rats were divided into the control group without LF supplementation and the lactoferrin group LF-supplemented (1000 mg/kg bw). (**A**) Effect of LF on the rat serum marker of bone formation, PINP. (**B**) Effect of LF on the rat serum marker of bone formation, BALP. (**C**) Effect of LF on the rat serum marker of bone resorption, CTX-1. (**D**) Effect of LF on the rat serum marker of bone resorption, TRACP. (**E**) Representative μ-CT images of proximal femur (scale bar = 2 mm). (**F**) Representative three-dimensional reconstructions μ-CT images of femoral trabeculae (scale bar = 1 mm). (**G**) Representative three-dimensional reconstructions μ-CT images of femoral cortex (scale bar = 2 mm). (**H**) Bone mineral density (BMD, g cm^−2^). (**I**) Bone volume/tissue volume (BV/TV, %). (**J**) Trabecular thickness (Tb.Th, mm). (**K**) Trabecular number (Tb.N, mm^−1^). (**L**) Trabecular separation/spacing (Tb.Sp, mm). (**M**) Cortical thickness (Ct.Th, mm). Data represent means ± SEM (* *p* < 0.05, ** *p* < 0.01, *n* = 10 per group). The control and lactoferrin groups were compared by an unpaired, two-tailed Student’s *t*-test. Each data point represents one rat.

The effects of lactoferrin supplementation on both bone formation and resorption in growing SD rats were evaluated by ELISA. The results show that LF-supplemented rats had a significantly higher PINP (*p* < 0.01; 13.69%; Figure 1A), significantly higher BALP (*p* < 0.01; 11.38%; Figure 1B), significantly lower CTX-1 (*p* < 0.01; 21.43%; Figure 1C), and significantly lower TRACP (*p* < 0.01; 41.67%; Figure 1D), compared with the control. LF increased markers of bone formation (PINP and BALP) and decreased markers of bone resorption (CTX-1 and TRACP). 

To assess the effects of LF on bone growth, μ-CT was used to measure femoral microarchitecture of growing SD rat. Features of the femoral bone (Figure 1E) and the microarchitecture of their trabeculae (Figure 1F) and cortices (Figure 1G) in the control and lactoferrin groups were illustrated. The femoral bone mineral density of the lactoferrin group was 2.8% (*p* < 0.05) higher than that of the control (Figure 1H). The microarchitecture of the trabeculae showed that BV/TV increased by 12.62% (Figure 1I), Tb.Th increased by 6.95% (Figure 1J) and Tb.N increased by 11.18% (Figure 1K) in the lactoferrin group (*p* < 0.05), compared with the control. Tb.Sp (Figure 1L) decreased by 22.22% (*p* < 0.05) compared with the control. For Ct.Th, however, LF had no significant influence (*p* > 0.05; Figure 1M), compared with the control.

### 3.2. LF Impacts on Osteogenesis by Upregulating Osteocalcin, Osterix, and Runx-2

LF supplementation improved bone status of growing SD rats by increasing bone formation and decreasing bone resorption. To explore the effects of LF supplementation on the bone formation in growing SD rats on cellular level, H&E staining and IHC analyses were used for determining the expression of osteogenesis markers (osteocalcin, osterix, and Runx-2) of osteogenic differentiation of osteoblasts in femur of growing SD rats.

**Figure 2:**
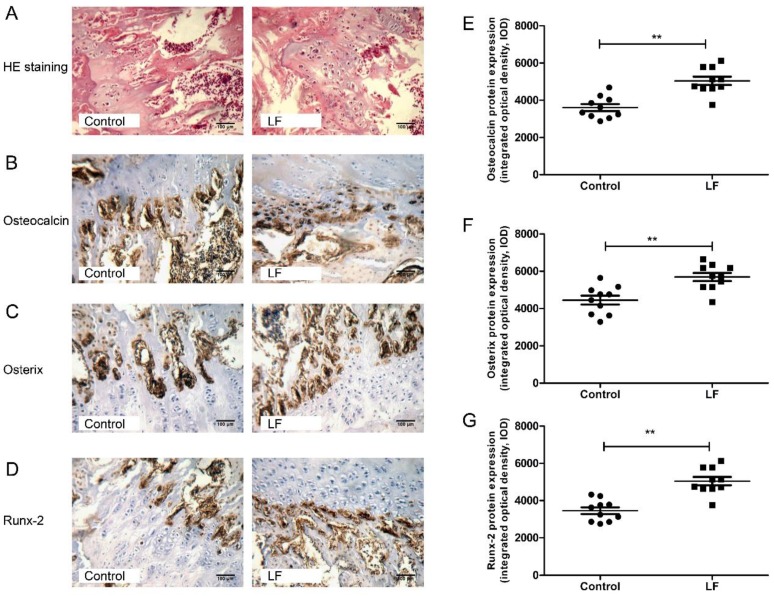
LF supplementation enhanced femoral bone formation of SD rat. (**A**) Representative photomicrographs of femur bone sections of SD rat strained with hematoxylin–eosin staining (×200 magnification, scale bar = 100 μm). (**B**–**D**) Representative images for the immunohistochemical staining detection of: osteocalcin (**B**); osterix (**C**); and Runx-2 (**D**). (×200 magnification, scale bar = 100 μm). (**E**–**G**) The integrated optical density (IOD) of target protein from immunohistochemistry was quantified by Image-Pro Plus software for: Osteocalcin (**E**); Osterix (**F**); and Runx-2 (**G**). Data are means ± SEM (** *p* < 0.01, *n* = 10 per group).

Figure 2A represents the representative photomicrographs of femur bone sections of SD rat strained with hematoxylin–eosin (H&E) staining. Several osteocytes and osteoblasts from the control group and LF group are clearly visible. The effects of LF on osteoblastogenesis markers were evaluated by IHC staining (Figure 2B–D). The integrated optical density (IOD) of target protein from IHC staining was quantified by IPP software. From osteocalcin, osterix, and Runx-2 of IHC analysis on femoral bone of growing SD rat, LF supplementation increased IOD value of target protein expression in osteogenic differentiation, osteocalcin levels by 40.71% (Figure 2E), osterix levels by 28.86% (Figure 2F), and Runx-2 levels by 47.42% (Figure 2G) relative to the control group. Overall, LF supplementation increased the expression of osteogenic differentiation proteinscompared with the control group. LF therefore promotes the osteogenic differentiation in vivo, which complements the results of our previous study in vitro [10].

### 3.3. Quality Assessment and Establishment of the Metabolomic Platform

**Figure 3:**
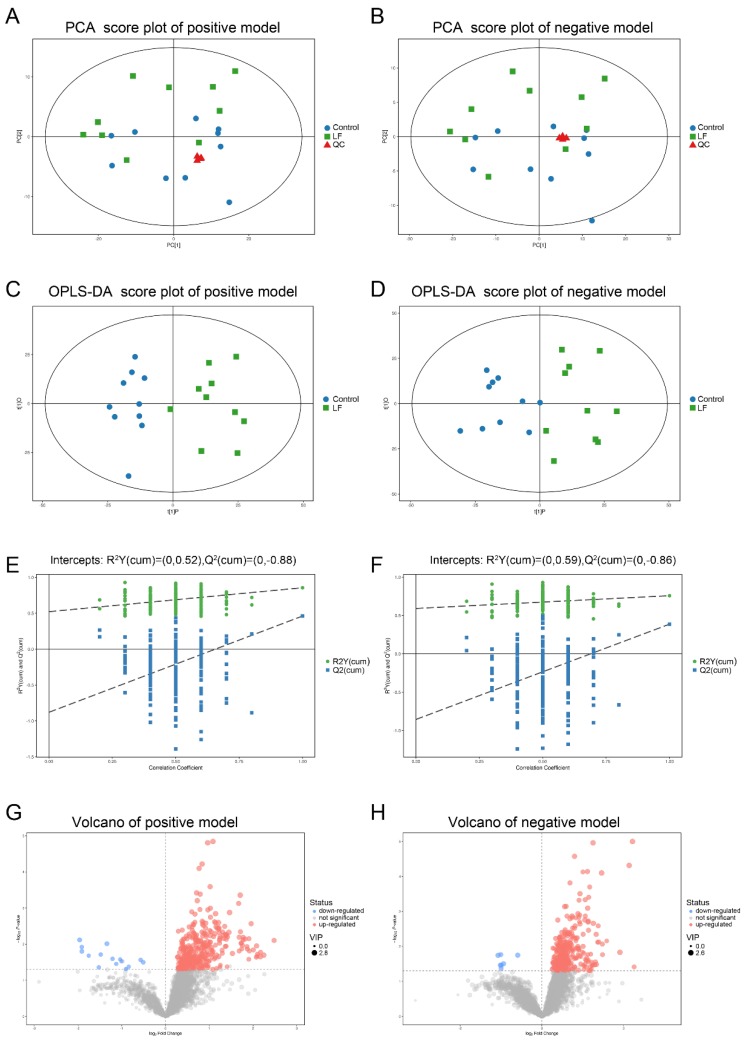
Multivariate analyses of urinary metabolic profiles of rats in the control and lactoferrin groups. (**A**,**B**) Principal component analysis (PCA) scores were plotted using positive (**A**) and negative (**B**) ion mode datasets for the control and lactoferrin groups and QC. PC[1], Component 1; PC[2], Component 2. Blue circles are the control group; green diamonds are the lactoferrin group; and red triangles are QC. (**C**,**D**) Orthogonal projections to latent structures–discriminate analysis (OPLS-DA) score plots of urinary metabolites in the control and lactoferrin groups obtained using positive (**C**) and negative (**D**) ion mode datasets. (**E**,**F**) Permutation test (*n* = 200) of OPLS-DA model in positive (**E**) and negative (**F**) ion modes. (**G**,**H**) Volcano plot of LF effects on urinary metabolites (*p* < 0.05; VIP > 1) in positive ((**G**), 370 metabolites) and negative ((**H**), 298 metabolites) ion modes.

LF supplementation improved bone growth and increased bone mass in growing SD rats. To determine whether the effects of LF on urinary metabolites were correlated with bone formation, urine samples of SD rat were analyzed by LC-MS/MS. Four representative pooled quality control (QC) samples by mixing equal volumes of urine samples from ten LF rats and ten control rats were prepared and analyzed in positive and negative ion modes in order to verify data reproducibility and reliability. The total ion chromatograms of the QC samples in the positive and negative ion modes are shown in Figure S1A,B. The extract ion chromatogram (EIC) of the 2-chloro-*L*-phenylalanine internal standard in the QC sample in positive and negative ion modes (Figure S2A,B) generated relative standard deviations (RSD) of 1.62% in positive ion mode, 1.92% in negative ion mode, the RSD < 20%, and QC sample correlation > 0.99. The PCA scores of the QC data were clustered in the middle of the score plot and separate from those for the experimental urine (Figure 3A,B) (red triangle). Our results show the metabolomic platform was very stable throughout the run and sufficient to ensure high data quality for subsequent global metabolomic analyses [40].

In total, 3881 peaks in positive ion mode and 3481 ion peaks in negative ion mode were detected by LC-MS/MS. After RSD de-noising, exclusion of metabolites with detection rates < 50%, filling in missing raw data with half the minimum value, and internal standard normalization, 3847 peaks in positive ion mode and 3459 peaks in negative ion mode were retained.

The unsupervised pattern recognition method and principal component analysis (PCA) were used to compare urine samples from the control and lactoferrin groups (Figure 3A,B). The PCA could not separate the two groups. To improve the resolution and identify the variables responsible for the segregation of these two groups, supervised orthogonal projections to latent structures–discriminant analysis (OPLS-DA) was applied. We obtained models for the positive ion mode (R^2^Y = 0.855; Q^2^ = 0.461) and the negative ion mode (R^2^Y = 0.758; Q^2^ = 0.387) that were stable, predictive, and that fit the data well. The models were valid, clearly discriminated the two groups, and demonstrated different metabolic profiles between them (Figure 3C,D). A permutation test validated these models (Figure 3E,F). Using R^2^Y = 0.52, and Q^2^ = −0.88 for the positive ion mode and R^2^Y = 0.59, Q^2^ = −0.86 for the negative ion mode, 200 permutations verified that the low Q intercept values indicated model robustness, low risks and lack of overfitting, and high reliability.

### 3.4. Metabolic Profiling Analysis of Urine from Growing SD Rats LF-Supplemented

The OPLA-DA score plots of the UPLC-MS/MS data were used to select the expression levels of various urinary metabolites affected by LF administration. Factors with variable importance in the projection (VIP) values > 1 were selected. These reflected the influences of each metabolite in both groups. Urinary metabolite concentrations were significantly different between groups according to Student’s *t*-test (*p* < 0.05). In the selected condition of VIP > 1, *p* < 0.05, LF altered the expressions of 370 metabolites (15 downregulated and 355 upregulated) in positive ion mode (Table S2) and 298 metabolites (7 downregulated and 291 upregulated) in negative ion mode (Table S3). The volcano plots (Figure 3G,H) show that LF upregulated most of the metabolites.

The metabolites were identified by searching the in-house MS2, KEGG, and HMDB databases. Based on the differentially expressed metabolites in the LF-supplemented SD rats, 69 metabolites were distinguished between two groups. Metabolites 1–37 were matched in positive ion mode and Metabolites 38–69 were matched in negative ion mode (Table 1). The endogenous rat urinary metabolites were significantly altered by lactoferrin.

### 3.5. Alterations in Metabolic Pathways Analysis

**Figure 4:**
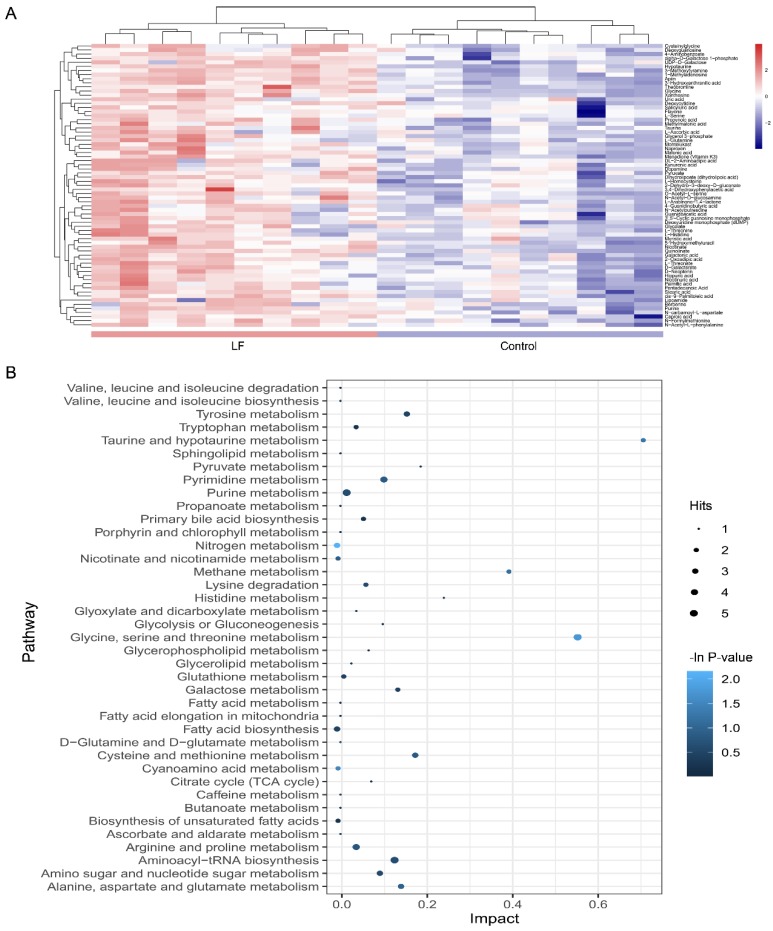
Metabolic pathway affected by LF supplementation. (**A**) Heatmap shows different expression levels of the 69 significantly altered metabolites in urine between the control and lactoferrin groups. Color range from blue to red represents metabolite level from low to high. (**B**) Bubble plot displays metabolic pathway enrichment analysis of the 69 significantly altered metabolites by LF supplementation. Node size is proportional with number of metabolites in given pathway and based on hits of each identified metabolite in a given pathway. Node color is graded according to its *p*-value in the pathway enrichment analysis.

A heatmap was generated to depict all metabolites in the dataset that were significantly altered and that matched in the HMDB and KEGG databases (Figure 4A). The heatmap demonstrated that LF changed the profile of urinary metabolites in vivo. A two-group cluster analysis could be separated; therefore, these metabolites were significantly affected by LF supplementation. Moreover, relative to the control group, there were higher urinary metabolite expression levels in the lactoferrin group. However, as our dataset included downregulated metabolites, it did not match the extracted MS/MS data. Thus, we assessed only the identified metabolites.

To clarify the effects of lactoferrin on the metabolic pathways, an enrichment analysis was performed on the metabolite pathways (Figure 4B). Among the 39 metabolic pathways identified, three were significantly enriched (*p* < 0.05; impact > 0.1). These findings demonstrate that three pathways were significantly disturbed in the lactoferrin group including nitrogen metabolism (*L*-glutamine cpd:C00064; glycine cpd:C00037; bs *L*-histidine cpd:C00135); glycine, serine, and threonine metabolism (*L*-serine cpd:C00065; glycine cpd:C00037; pyruvic acid cpd:C00022; guanidoacetic acid cpd:C00581; and *L*-threonine cpd:C00188); and cyanoamino acid metabolism (glycine cpd:C00037 and *L*-serine cpd:C00065).

### 3.6. Correlation between Urinary Metabolites and Bone Growth Induced by LF Supplementation

**Figure 5:**
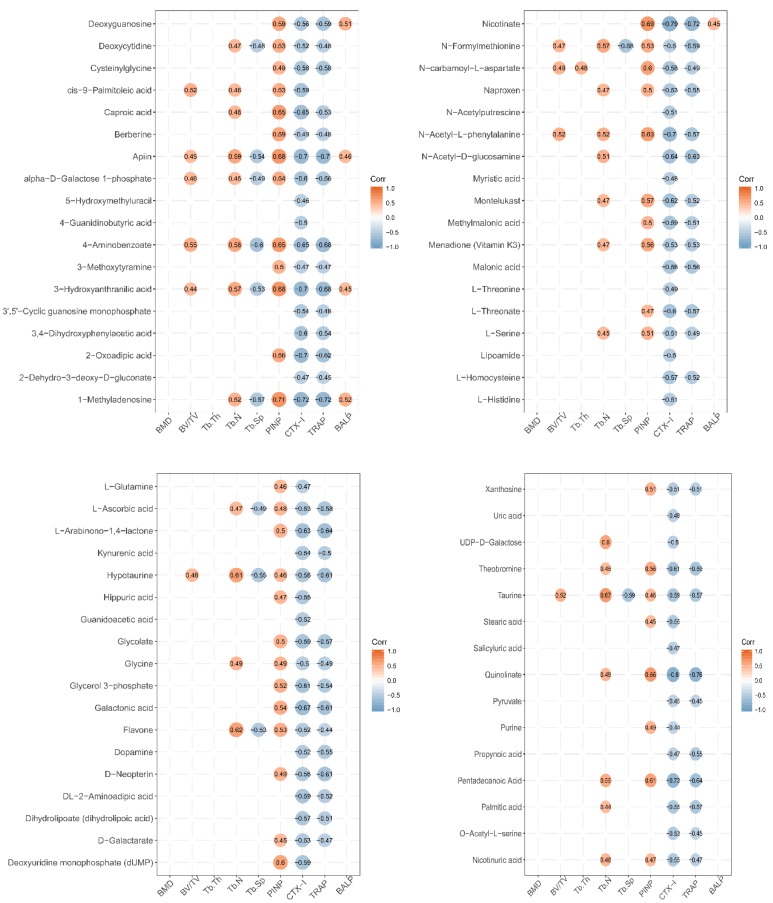
Correlation between expression level of urine metabolites and bone status in LF-supplemented rats. Pearson correlation analysis was used to assess the relationship between expression levels of urine metabolites and bone status (CTX-1, TRACP, PINP, BALP, BMD, BV/TV, Tb.Th, Tb.Sp, and Tb.N). Pearson correlation is significantly different at a level of 0.05, and they are not shown in the graph when *p* ≥ 0.05. Red circle represents positive correlation, and blue circle represents negative correlation. The value in circle represents Pearson correlation coefficient *r*.

To establish whether the significantly altered metabolite levels were associated with bone growth, a Person’s correlation coefficient analysis was performed using bone-related markers and urinary metabolites, and all group sample data were pooled (Figure 5).

Several metabolites (caproic acid, apiin, 4-aminobenzoate, 3-hydroxyanthranilic acid, 1-methyladenosine, nicotinate, n-carbamoyl-l-aspartate, n-acety-l-phenylalanine, deoxyuridine monophosphate (dump), quinolinate, and pentadecanoic acid) were significantly positively correlated with the markers of bone formation PINP (Person’s correlation coefficient *r* > 0.6; *p* < 0.05). Several metabolites (caproic acid, apiin, alpha-d-galactose 1-phosphate, 4-aminobenzoate, 3-hydroxyanthranilic acid, 3,4-dihydroxyphenylacetic acid, 2-oxadipic acid, 1-methyladenosine, nicotinate, n-acetyl-d-glucosamine, montelukast, l-threonate, glycerol 3-phosphate, galactonic acid, theobromine, quinolinate, and pentadecanoic acid) were significantly negatively correlated with the markers of bone resorption, CTX-1 (*r* > 0.6; *p* < 0.05). Several metabolites (apiin, 4-aminobenzoate, 2-oxoadipic acid, 1-methyladenosine, nicotinate, n-acetyl-d-glucosamine, l-arabinono-1,4-lactone, hypotaurine, galactonic acid, d-neopterin, quinolinate, and pentadecanoic acid) were significantly negatively correlated with the markers of bone resorption, TRACP (*r* > 0.6; *p* < 0.05). However, the urine metabolites were not significantly correlated with BMD, BV/TV, Tb.Th, Tb.N, Tb.Sp, and BALP (*r* < 0.6 or *p* > 0.05).

## 4. Discussion

The effects of LF supplementation on bone status of growing SD rat were analyzed via measuring the associated regulatory factors in the serum by ELISA, bone mass by μ-CT measure, and osteogenesis marker by IHC analysis. The urinary metabolite profiles of SD rat LF-supplemented (1000 mg/kg bw) for four weeks were analyzed by LC-MS/MS. We aimed to explore the urinary metabolic profiles of LF-supplemented SD rats by LC-MS/MS, and to elucidate the metabolic pathway related to bone status, and to further explore the possible mechanisms by which LF impacts on bone formation in growing SD rats at the metabolic level in vivo.

LF plays a key role in promoting bone health. LF supplementation enhances bone mass in ovariectomized mice by modulating bone formation and resorption as well as modulating immune function in the bone microenvironment [17,41]. In the present study, we found that LF supplementation in growing rats elevated bone mass by increasing the serum levels of the bone formation markers PINP and BALP, and by decreasing the serum levels of the bone resorption markers CTX-1 and TRACP. These findings corroborate those reported by Li et al. [42]. LF significantly improves bone regeneration by increasing the bone volume fraction (BV/TV) in a rat calvarial defect model [13]. It also stimulates increases in the volume and density of new bone formed in midpalatal sutures during rapid palatal expansion [43]. Our study showed that LF supplementation increased femoral bone mass and femoral trabecular microarchitecture. LF significantly increased bone mineral density (BMD), BV/TV, Tb.Th, and Tb.N (*p* < 0.05) and significantly decreased Tb.Sp (*p* < 0.05), but did not significantly influence thickness (*p* > 0.05). Our findings are consistent with the results of Fan et al. [17]. Thus, LF supplementation improved bone mass of SD rat during the growing period. LF supplementation improved the bone status of growing SD rats by increasing bone formation and decreasing bone resorption.

LF stimulates osteogenic differentiation of osteoblast [18] and stem cell (osteoblast precursor cells) [44] in vitro. In growing SD rats, bone marrow comprises a number of bone marrow stem cells which may differentiate into osteoblasts in specific bone microenvironments. LF promotes osteogenic differentiation of bone marrow mesenchymal stem cells [10,45]. In our study, IHC of osteogenic differentiation markers revealed significant upregulation expression of osteocalcin, osterix, and Runx-2 in SD rat femur following LF supplementation compared with the control group. Runx-2 is a key transcription factor associated with osteoblast differentiation, which participates in osteogenesis, and regulates the transcription of downstream osteogenic genes such as alkaline phosphatase, collagen type-1, and osteocalcin [46]. Otherwise, LF could stimulate the expression of osteocalcin and Runx-2 [11,47]. Osterix is an osteoblast-specific transcription factor associated with osteoblast differentiation and bone formation [48]. Montesi et al. showed that LF can increase osterix expression in osteoblasts [49]. Our results also confirm that LF supplementation in SD rats improved the osteoblasts osteogenic in bone formation by increasing the expression of osteocalcin, osterix, and Runx-2.

The absorption, metabolism, and disposal of nutrients can affect biological functions, thereby preventing disease and maintaining health. The effects of LF on urine metabolic profiles in growing SD rats receiving LF for four weeks were evaluated by LC-MS/MS and multivariate analysis. Metabolic profiles may be run on a wide range of metabolites in cells, tissues, organs, or organisms [24]. Non-targeted metabolomics was used to investigate differentially expressed metabolites in urine samples. In total, 69 urine metabolites of SD rats that were significantly affected by LF supplementation were identified. KEGG database was searched to identify the pathways in which the various metabolites were involved. Those metabolites related to bone metabolism included taurine and hypotaurine metabolism; arginine and proline metabolism; ascorbate and aldarate metabolism; cyanoamino acid metabolism; glycine, serine, and threonine metabolism; nitrogen metabolism; nicotinate and nicotinamide metabolism; and fatty acid biosynthesis (Figure 4B).

Urinalysis disclosed that the metabolic pathways of taurine and hypotaurine were affected by LF supplementation. LF significantly increased urinary taurine and hypotaurine levels relative to the control group (Table 1). The taurine metabolic pathway was associated with bone demineralization in premenopausal and postmenopausal Chinese women. Thus, reduced taurine is a potential biomarker of osteoporosis [50]. The results of correlation between taurine and the marker of bone formation and resorption showed that the expression of taurine was positive to PINP and negative to CTX-1 and TRACP; the Pearson correlation coefficients were 0.46, −0.59, and −0.57, respectively (Figure 5). The Pearson correlation coefficients between hypotaurine and the marker of bone status were 0.46 (PINP), −0.56 (CTX-1), −0.61 (TRACP), and 0.61 (Tb.N) (Figure 5). These results demonstrate that taurine and hypotaurine were affected by LF, and were related to bone formation and resorption.

LF supplementation altered arginine and proline metabolism. Kotwal et al. found that supplementation of calcium, vitamin D, *L*-lysine, *L*-proline, *L*-arginine, and *L*-ascorbic acid promotes bone mineralization in ovariectomized rats [51]. Elevated proline, arginine, glutamine, and dipeptides are found in the osteonecrotic and adjacent “normal” bone trabeculae of human femoral head patients [52]. In this study, L-glutamine and guanidoacetic acid levels were higher in the lactoferrin group than the control group. The Pearson correlation coefficients between L-glutamine and PINP (the marker of bone formation) and between L-glutamine and CTX-1 (the marker of bone resorption) were 0.46 and −0.47, respectively (Figure 5). The Pearson correlation coefficient between guanidoacetic acid and CTX-1 was −0.52. Thus, LF supplementation can change arginine and proline metabolism, and these metabolites were related to bone formation and bone resorption. Li et al. reported that pro-hydroxy-pro BCAA levels in the plasma of neonatal piglets supplemented by lactoferrin are associated with enhanced bone status because they modulate bone formation [42]. Therefore, arginine and proline metabolism, which regulate bone metabolites, may be affected by LF supplementation. Otherwise, *L*-ascorbic acid increased in the urine after LF supplementation in our study. *L*-ascorbic acid is a bioactive factor that may participate in cell differentiation [53]. It can promote osteoblast formation and block osteoclastogenesis via Wnt/β-catenin/ATF4 signaling pathways [54].

Cyanoamino acid metabolism and glycine, serine, and threonine metabolism (glycine, *L*-serine, and pyruvic acid) were affected by lactoferrin supplementation. Serine is a pyruvate precursor. In premenopausal women, low Gly-Gly levels are associated with low BMD [55]. Serine and pyruvate levels of SD rat urine in the lactoferrin group significantly increased, compared with those of the control group. Glycine was associated with PINP (*r* = 0.49), Tb.N (*r* = 0.49), CTX-1 (*r* = −0.5), and TRACP (*r* = −0.49); L-serine was associated with PINP (*r* = 0.51), Tb.N (*r* = 0.45), CTX-1 (*r* = −0.51), and TRACP (*r* = −0.49); and pyruvate was associated with CTX-1 (*r* = −0.45) and TRACP (*r* = −0.45). These results show that LF supplementation affected the cyanoamino acid metabolism and glycine, serine, and threonine metabolism in SD rat, which metabolic pathways were related to bone formation and bone resorption.

Nitrogen metabolism (*L*-glutamine and *L*-histidine) was altered in our urinary metabolic profile. Skeletal stem cells upregulate glutamine metabolism during osteoblast differentiation [56]. The metabolism of glutamine and other essential nutrients are interrelated and participate in differentiation of bone marrow-derived mesenchymal stem cell [57]. Glutaminase deaminates glutamine to glutamate and is the rate-limiting step in glutamine metabolism [58]. Here, L-histidine was correlated with CTX-1(*r* = −0.51) and L-glutamine was correlated with PINP (*r* = 0.46) and CTX-1(*r* = −0.47). Thus, nitrogen metabolism was affected by LF supplementation, and it may be related to bone metabolism.

Nicotinate and nicotinamide metabolism (quinolinic acid and nicotinic acid) was affected by lactoferrin supplementation in SD rats. Nicotinic acid may be converted to nicotinamide which participates in NAD or NADP synthesis. Therefore, nicotinate and nicotinamide metabolism plays key roles in vital regulatory functions [59]. In this study, the expression of quinolinate was correlated with PINP (*r* = 0.66), CTX-1 (*r* = −0.8), and TRACP (*r* = −0.76), while the expression of nicotinic acid was correlated with PINP (*r* = 0.47), CTX-1 (*r* = −0.55), and TRACP (*r* = −0.47). Thus, nicotinate and nicotinamide metabolism was affected by LF supplementation, and it may be related to bone metabolism.

Fatty acid (myristic acid, stearic acid, and palmitic acid) biosynthesis was affected by LF supplementation in SD rats. Fatty acids are important components of cell membrane and can affect bone metabolism by regulating calcium absorption, osteoclast formation, prostaglandins synthesis, cell membrane n-6/n-3 fatty acid ratios, cytokine levels, and lipid peroxidation. Osteoblasts require adequate fatty acid levels to maintain skeletal mass [60]. In this study, the expression of myristic acid was associated with CTX-1 (*r* = −0.48); the expression of stearic acid was associated with PINP (*r* = 0.45); and the expression of palmitic acid was associated with CTX-1 (*r* = −0.55), TRACP (*r* = −0.57), and Tb.N (*r* = 0.44).

Bone formation regulation is complex and still poorly understood, which includes comprehensive pathways. Metabolomics may provide global, unbiased metabolite-based biological system profiles that differ from those obtained by genomics, transcriptomics, and proteomics. All small-molecule metabolites, which were generated in a cell, tissue, organ, or organism, were compiled and globally analyzed by metabolomics. These metabolites represent the sum total of all metabolic pathways in an organism. This information may be used to identify each metabolic pathway and its role in the metabolic functions of an organism. The changed metabolites in urine can represent the results of all the effects after the body is affected by the environment. Therefore, we can explore the effects of nutrient supplementation in vivo by studying urinary metabolic profiling. The in vivo effects of LF supplementation are complex. In sum, LF upregulated 69 urinary metabolites of growing rats. KEGG and pathway enrichment analyses of those urinary metabolites, and the Person’s correlation analysis among those urinary metabolites and bone status revealed that LF impacted on bone formation via regulatory comprehensive pathways including taurine and hypotaurine metabolism, arginine and proline metabolism, cyanoamino acid metabolism, nitrogen metabolism, nicotinate and nicotinamide metabolism, and fatty acid biosynthesis. The Person’s correlation coefficient between the single urinary metabolite and bone status was not high, but it was significant (*p* < 0.05) (Figure 5). This invariably indicated that LF promoted bone formation via regulating not one pathway, but comprehensive pathways in vivo. LF may improve bone mass and immunological development in neonates [61,62] and significantly alter the intestinal microbiome [63,64]. The improvements in bone status realized by LF supplementation may involve alterations in the levels of numerous metabolites and multiple metabolic pathways. For these reasons, further investigation is warranted here. The regulatory mechanisms of bone formation and resorption also control the metabolism of global energy [65], glucose [66], and fatty acids [60]. Thus, studying the mechanism by which LF promotes bone health in metabolic level is valuable.

There are some limitations of our current study. Integration with other omics platforms, such as proteomics and transcriptomics, would provide a more comprehensive snapshot of the biological impact of LF supplementation. Otherwise, LF supplementation also alters intestinal immunity [67] and alters intestinal flora [68,69], which may be related to bone health. Future work should include multi-omics validation of these results, focusing on changed metabolites which are related to bone formation in vivo and in vitro.

The present study applied a powerful metabolomic pipeline based on liquid chromatography coupled to high-resolution mass spectrometry along with multivariate statistics and pathway analysis to identify the effects of lactoferrin on bone formation. Our study indicated that the metabolomics is a useful and practical tool to investigate the mechanism for nutritional supplementation. This study laid the foundation for further research into the mechanisms by which LF promotes bone health.

## 5. Conclusions

It was found that lactoferrin supplementation (1000 mg/kg bw) improved bone formation by upregulating serum PINP and BALP (*p* < 0.05) and diminished bone resorption by downregulating CTX-1 and TRACP (*p* < 0.05). Lactoferrin also improved trabecular microarchitecture (*p* < 0.05) and upregulated the osteogenic markers of osteocalcin, osterix, and Runx-2 (*p* < 0.05) during bone formation. Lactoferrin upregulated 69 urinary metabolites determined by LC-MS/MS based on metabolomic. KEGG and pathway enrichment analyses of those urinary metabolites, and the Person’s correlation analyses among those urinary metabolites and bone status showed LF impacted on bone formation via regulatory comprehensive pathways including taurine and hypotaurine metabolism, arginine and proline metabolism, cyanoamino acid metabolism, nitrogen metabolism, nicotinate and nicotinamide metabolism, and fatty acid biosynthesis. This work demonstrated that lactoferrin supplementation altered numerous metabolic pathways result in improving bone mass during the growth period. It also indicated the metabolomics is a useful and practical tool to explore the mechanisms by which LF augments bone mass formation in growing animals.

## Figures and Tables

**Table 1:** Significantly different metabolites in the urine of SD rats in the control and lactoferrin groups.

No.	Metabolites Name	*m/z*	Exact Mass	RT (min)	Fold Change (LF/Control)	Sub.Class
1	3,4-Dihydroxyphenylacetic acid	359.067	168.042	5.429	1.315	Phenols
2	3-Hydroxyanthranilic acid	154.050	153.043	3.508	2.188	Benzoic acids and derivatives
3	3-Methoxytyramine	234.042	167.095	2.322	1.464	Phenols
4	4-Aminobenzoate	160.039	137.048	3.150	1.892	Benzene and substituted derivatives
5	4-Guanidinobutyric acid	146.092	145.085	5.682	1.471	Carboxylic acids and derivatives
6	alpha-D-Galactose 1-phosphate	225.015	260.030	3.939	1.529	Organooxygen compounds
7	Apiin	529.139	564.148	3.861	2.209	
8	Berberine	413.053	336.124	5.052	2.373	Protoberberine alkaloids and derivatives
9	Caproic acid	183.039	116.084	2.952	1.593	Fatty Acyls
10	cis-9-Palmitoleic acid	272.258	254.225	1.114	2.606	Fatty Acyls
11	Cysteinylglycine	223.018	178.041	5.694	1.341	Carboxylic acids and derivatives
12	Deoxyguanosine	267.097	267.097	4.080	1.818	Purine nucleosides
13	D-Galactarate	228.068	210.038	5.777	1.392	Organooxygen compounds
14	Dihydrolipoate (dihydrolipoic acid)	208.063	208.059	4.757	2.017	Fatty Acyls
15	DL-2-Aminoadipic acid	144.064	161.069	4.751	1.674	Carboxylic acids and derivatives
16	Dopamine	136.075	153.079	4.752	1.522	Benzenediols
17	Flavone	223.074	222.068	4.565	1.574	Flavones
18	Glycerol 3-phosphate	345.035	172.014	5.203	1.298	Glycerophospholipids
19	Guanidoacetic acid	118.061	117.054	5.565	1.477	Amino acids, peptides, and alogues
20	Hypotaurine	110.026	109.020	5.442	2.210	Sulfinic acids and derivatives
21	Kynurenic acid	190.052	189.043	4.751	1.890	Quinolines and derivatives
22	L-Histidine	156.076	155.069	4.959	1.465	Amino acids, peptides, and alogues
23	L-Threonine	84.044	119.058	4.908	1.298	Carboxylic acids and derivatives
24	Malonic acid	146.044	104.011	7.055	1.951	Dicarboxylic acids and derivatives
25	Montelukast	603.245	585.210	6.349	1.404	
26	Myristic acid	246.242	228.209	1.060	1.651	Fatty acids and conjugates
27	N-Acetyl-D-glucosamine	222.096	221.090	5.509	1.304	Organooxygen compounds
28	N-Acetylputrescine	131.117	130.111	5.396	1.337	Carboximidic acids and derivatives
29	Naproxen	272.134	230.094	6.131	1.394	
30	N-carbamoyl-L-aspartate	237.074	176.043	5.530	1.324	Carboxylic acids and derivatives
31	O-Acetyl-L-serine	148.060	147.053	5.509	1.272	Amino acids, peptides, and alogues
32	Palmitic acid	274.274	256.240	0.910	1.968	Fatty acids and conjugates
33	Pentadecanoic Acid	260.257	242.225	0.964	2.027	Fatty Acyls
34	Propynoic acid	141.017	70.005	0.721	1.301	Carboxylic acids and derivatives
35	Purine	263.074	120.044	1.970	1.320	
36	Stearic acid	302.304	284.272	0.876	2.451	Fatty acids and conjugates
37	UDP-D-Galactose	567.052	566.055	2.043	1.607	Pyrimidine nucleosides
38	1-Methyladenosine	262.091	281.112	3.884	2.210	
39	2-Dehydro-3-deoxy-D-gluconate	177.040	178.048	4.918	1.499	Keto acids and derivatives
40	2-Oxoadipic acid	159.029	160.037	5.704	1.668	Keto acids and derivatives
41	3′,5′-Cyclic guanosine monophosphate	344.038	345.047	5.033	1.486	
42	5-Hydroxymethyluracil	158.053	142.038	5.955	1.275	Diazines
43	Deoxycytidine	226.083	227.091	3.293	1.449	Pyrimidine nucleotides
44	Deoxyuridine monophosphate (dUMP)	307.030	308.041	0.619	1.408	Pyrimidine nucleosides
45	D-Neopterin	312.093	253.081	5.424	1.352	Pteridines and derivatives
46	Galactonic acid	195.051	196.058	5.715	1.608	Hydroxy acids and derivatives
47	Glycine	74.025	75.032	4.341	1.320	Amino acids, peptides, and alogues
48	Glycolate	75.009	76.016	5.032	1.286	Hydroxy acids and derivatives
49	Hippuric acid	178.051	179.058	3.150	1.458	Benzamides
50	L-Arabinono-1,4-lactone	207.125	148.114	5.082	1.516	
51	L-Ascorbic acid	175.025	176.032	0.716	1.218	Furanones
52	L-Glutamine	145.061	146.069	4.897	1.324	Carboxylic acids and derivatives
53	L-Homocysteine	269.069	135.035	5.363	1.387	Carboxylic acids and derivatives
54	Lipoamide	221.083	205.060	2.891	1.531	Dithiolanes
55	L-Serine	104.035	105.043	4.713	1.396	Amino acids, peptides, and alogues
56	L-Threonate	135.030	136.037	5.029	1.349	Organooxygen compounds
57	Menadione (Vitamin K3)	343.102	172.052	4.616	1.476	phthoquinones
58	Methylmalonic acid	117.019	118.027	1.388	1.359	Dicarboxylic acids and derivatives
59	N-Acetyl-L-phenylalanine	206.082	207.090	2.960	1.672	Carboxylic acids and derivatives
60	N-Formylmethionine	176.038	177.046	3.267	1.526	Carboxylic acids and derivatives
61	Nicotinate	122.024	123.032	6.842	1.608	Pyridinecarboxylic acids and derivatives
62	Nicotinuric acid	180.056	180.053	3.150	1.455	Carboxylic acids and derivatives
63	Pyruvate	175.025	88.016	5.009	1.348	Alpha
64	Quinolinate	166.014	167.022	6.403	1.737	Pyridines and derivatives
65	Salicyluric acid	194.047	195.053	4.692	1.354	Benzene and substituted derivatives
66	Taurine	124.008	125.015	4.668	1.549	Organosulfonic acids and derivatives
67	Theobromine	180.066	180.065	3.501	1.661	Purines and purine derivatives
68	Uric acid	167.020	168.028	3.128	1.342	Purines and purine derivatives
69	Xanthosine	283.067	284.076	4.891	1.301	Purine nucleosides

## Supplementary Materials

The following are available online at https://www.mdpi.com/2072-6643/12/4/1116/s1, Figure S1: The total ion chromatogram of QC samples. (A) The total ion chromatogram of QC in positive ion mode. (B) The total ion chromatogram of QC in negative ion mode. Figure S2: The extract ion chromatogram (EIC) of internal standard 2-Chloro-L-phenylalanine in QC sample. (A) The EIC of2-Chloro-L-phenylalanine in positive ion mode. (B) The EIC of 2-Chloro-L-phenylalanine in negative ion mode. Table S1: The condition of mobile phase in UHPLC system. Table S2: The metabolites of urine affected by LF in positive model by LC-MS/MS analysis. The metabolites were selected by the condition of VIP > 1, *p* < 0.05. Table S3: The metabolites of urine affected by LF in negative model by LC-MS/MS analysis. The metabolites were selected by the condition of VIP > 1, *p* < 0.05.

## Abbreviations

BALP, bone alkaline phosphatase; BMD, bone mineral density; Ct.Th, cortical thickness; CTX-1, carboxyl-terminal telopeptide of type 1 collagen; EIC, extract ion chromatogram; ELISA, enzyme-linked immunosorbent assay; ESI, electrospray ionization; H&E, hematoxylin–eosin staining, HPLC, high-performance liquid chromatography; IHC, immunohistochemistry; KEGG, Kyoto Encyclopedia of Genes and Genomes; LC-MS/MS, liquid chromatography-tandem mass spectrometry; LF, lactoferrin; *m/z*, mass-to-charge ratio; μ-CT, microcomputerized tomography; OPLS-DA, orthogonal projections to latent structures–discriminant analysis; PBS, phosphate-buffered saline; PCA, principal components analysis; PINP, procollagen type I *N*-terminal propeptide; QC, quality control; ROI, region of interest; RSD, relative standard deviations; RT, retention time; Runx-2, Runt-related transcription factor; SD, Sprague-Dawley; Tb.N., trabecular number; Tb.Sp., trabecular spacing; Tb.Th., trabecular thickness; TRACP, tartrate-resistant acid phosphatase; UHPLC-QTOF-MS, ultra-high performance liquid chromatography/quadrupole time-of-flight mass spectrometry; VIP, variable influence on projection.
