# Supplementary material for: Urinary Metabolic Profiling via LC-MS/MS Reveals Impact of Bovine Lactoferrin on Bone Formation in Growing SD Rats
Source: Nutrients. 2020 Apr 17;12(4):1116. doi: 10.3390/nu12041116 (PMC7230685; doi:10.3390/nu12041116)
Supplement: Supplementary file 1 [file nutrients-12-01116-s001.pdf]

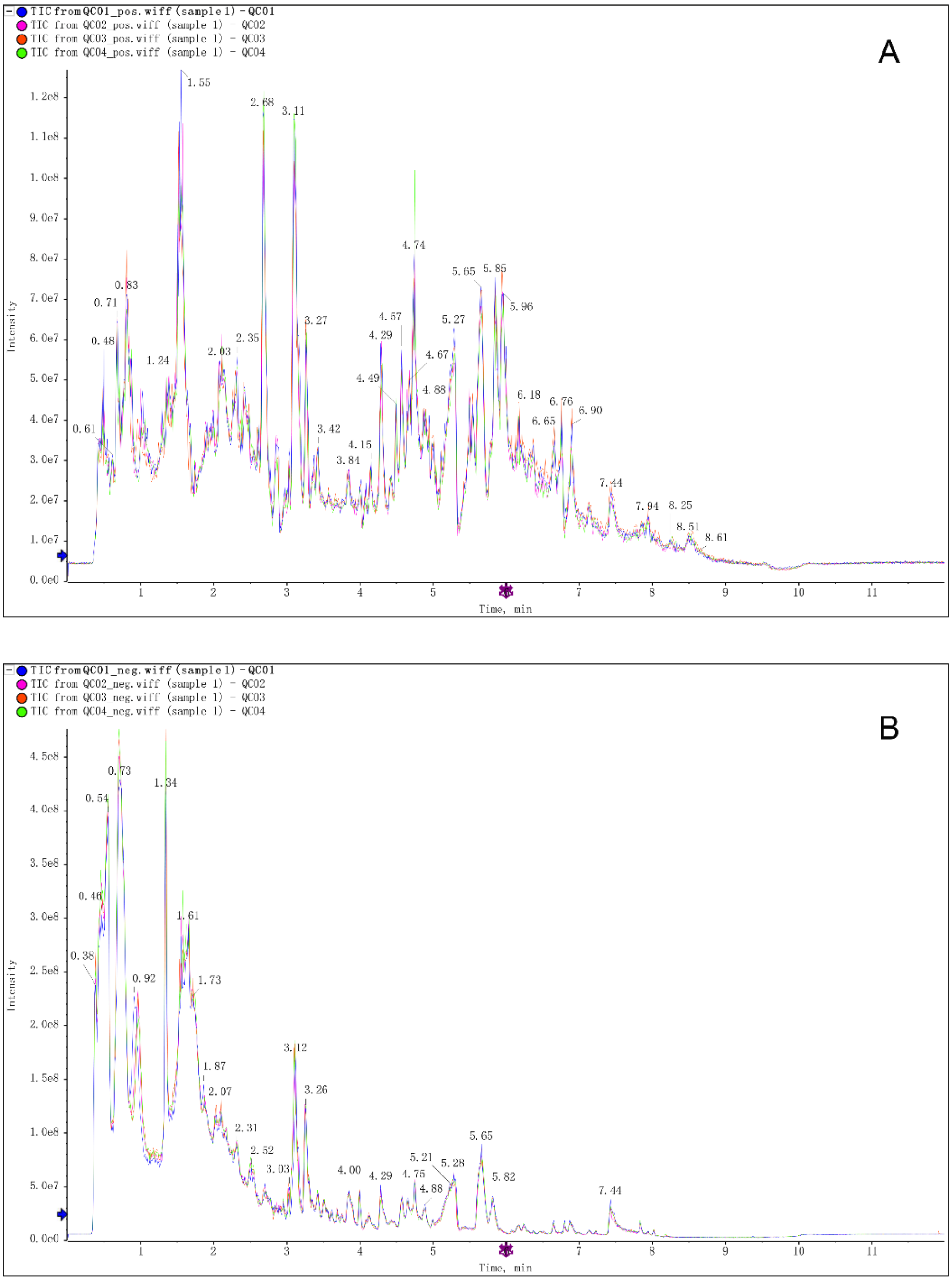

Figure S1. The total ion chromatogram of QC samples: (**A**) the total ion chromatogram of QC in positive ion mode; and (**B**) the total ion chromatogram of QC in negative ion mode.

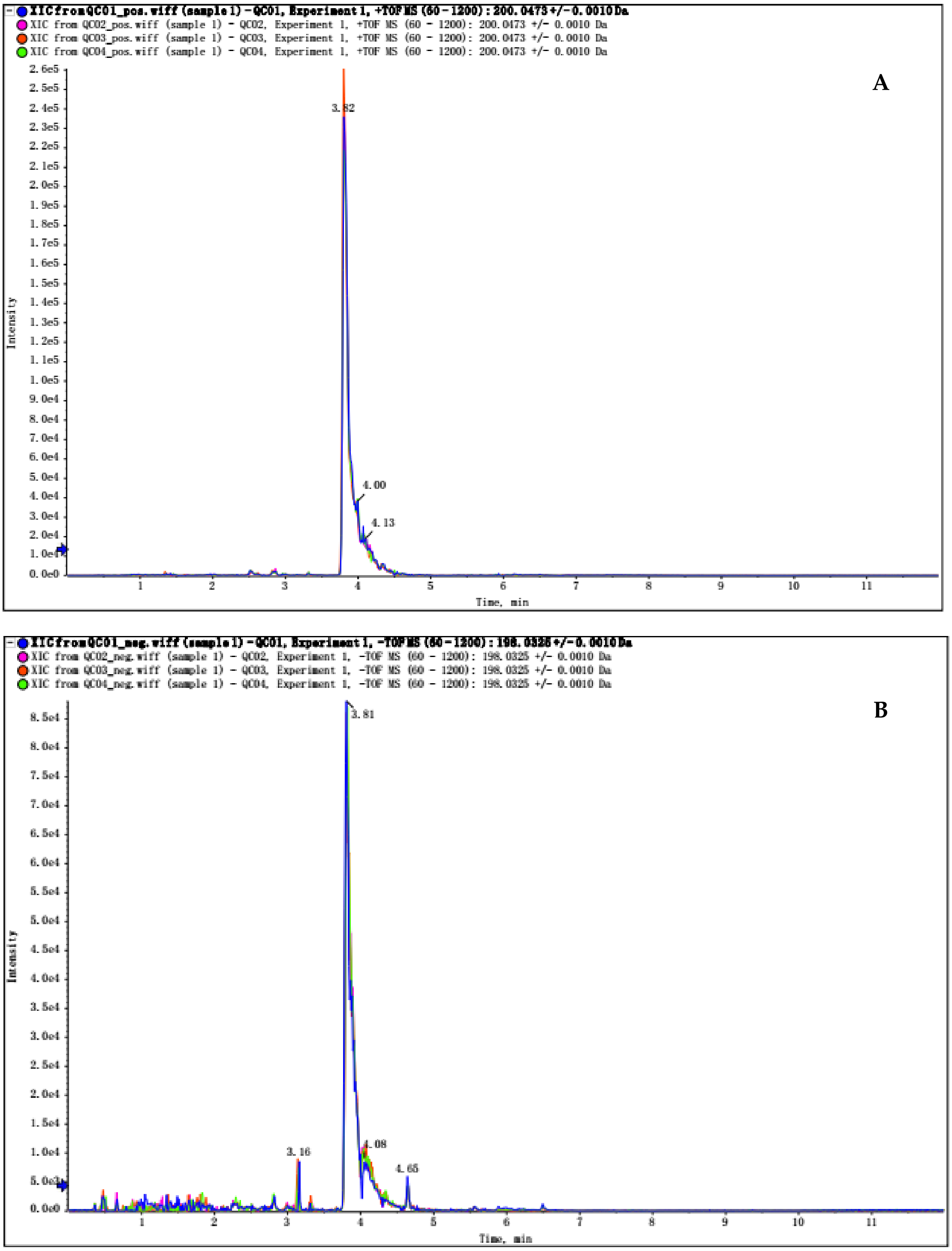

Figure S2. The extract ion chromatogram (EIC) of internal standard 2-Chloro-L-phenylalanine in QC sample: (**A**) the EIC of 2-Chloro-L-phenylalanine in positive ion mode; and (**B**) the EIC of 2-Chloro-L-phenylalanine in negative ion mode.

Table S1. The condition of mobile phase in UHPLC system.

Time (min)	The Flow Rate (μL/min)	A% (25 mM NH_4Ac, 25 mM NH_4OH)	B% Acetonitrile
0	500	5	95
0.5	500	5	95
7	500	35	65
8	500	60	40
9	500	60	40
9.1	500	5	95
12	500	5	95

Table S2. The differentially expressed urine metabolites in positive mode by LC-MS/MS analysis.

No.	mz	rt	MEAN LF	MEAN CONTROL	VIP	*p*-VALUE	Q-VALUE	FOLD CHANGE	LOG_FOLDCHANGE
1	84.0439391	294.491	0.417087463	0.32134115	1.593289937	0.044330672	0.411554337	1.297958455	0.376244206
2	101.0339442	333.8905	0.085992153	0.058704836	1.03974747	0.028518878	0.350785559	1.464822304	0.550725664
3	101.1065659	114.339	0.386091761	0.113000569	2.39086263	0.006534976	0.278381866	3.416724051	1.772613734
4	102.1275262	623.816	1.048455843	0.788664426	1.810702193	0.03203568	0.361427942	1.32940679	0.410782628
5	110.0263269	326.518	0.032353437	0.014637917	2.437383987	0.002560231	0.262119641	2.210248754	1.144208748
6	110.0704506	297.585	0.330423622	0.226155326	1.449224727	0.028178532	0.350056089	1.461047276	0.547002861
7	112.0749654	273.916	0.539118773	0.423215675	1.948974362	0.034510866	0.371934533	1.27386296	0.349210084
8	118.0609417	333.909	1.429293466	0.967632938	1.031783958	0.025407286	0.343526951	1.477102949	0.56277038
9	119.0848403	36.81	0.163531331	0.116536125	1.232943168	0.034041617	0.369789873	1.403267283	0.488789828
10	124.0495583	174.6815	0.117607706	0.090538979	2.016576637	0.027127123	0.347707752	1.298973183	0.377371647
11	126.0208611	297.906	0.201069933	0.150620211	1.280197307	0.042476402	0.404936577	1.334946564	0.416781994
12	129.1380606	63.611	0.875548483	0.554471989	1.482533625	0.030397791	0.354998789	1.579067113	0.659072489
13	130.1583017	145.592	0.141700599	0.095539041	2.212005341	0.016258989	0.31040356	1.483169569	0.568683549
14	131.1172007	323.7895	0.116257922	0.08692258	1.321212103	0.032345343	0.362594711	1.337488173	0.419526135
15	136.0746076	285.097	0.044212894	0.029047776	1.672997816	0.02694109	0.347276611	1.52207503	0.606039477
16	141.0172295	43.2695	0.139654162	0.107370612	1.544547478	0.043886174	0.409999717	1.300673978	0.379259387
17	144.0436711	184.628	1.233570545	0.773483644	2.202571045	0.001471712	0.244734349	1.594824344	0.673397532
18	144.0644986	285.061	0.224588666	0.134192931	1.519909978	0.009107684	0.285066707	1.673625166	0.742976451
19	144.0813751	170.512	0.181363267	0.090057797	1.479287065	0.028579716	0.350914439	2.013854133	1.00995919
20	146.0437092	423.303	0.102246868	0.052418791	2.099333603	0.044290531	0.41141475	1.950576599	0.963900654
21	146.0924715	340.9315	7.160504738	4.867528337	1.128235094	0.037485483	0.385219572	1.471076128	0.556871908
22	147.0755003	356.5795	0.240537309	0.19965691	1.726952553	0.022828435	0.336321419	1.204753242	0.268737684
23	148.059611	330.523	0.06579105	0.051703836	1.657777248	0.030197941	0.354183266	1.272459746	0.347620017
24	152.0806426	297.4845	0.15144845	0.105938409	1.281947341	0.041656392	0.401895234	1.429589621	0.515601065
25	154.0495139	210.473	0.315047624	0.144012581	2.517442533	0.00513466	0.275380644	2.187639599	1.129375082
26	155.0805432	275.252	0.79129321	0.47645661	1.63216024	0.030152636	0.354095738	1.660787559	0.731867541
27	156.0756294	297.5545	0.190385973	0.129923574	1.468110155	0.032218353	0.362118034	1.465368964	0.551263966
28	157.0752901	231.712	0.103923323	0.037679357	2.752518968	0.001329566	0.240719571	2.758097022	1.463673208
29	160.0388299	188.9845	0.036111905	0.019088427	2.240750127	0.002620196	0.262697062	1.891821961	0.919776323
30	160.1072022	293.201	0.393681724	0.301857053	1.80413835	0.009394265	0.28604086	1.304199189	0.383164227
31	160.1073417	349.4445	0.750532807	0.587427879	1.29290704	0.0224347	0.335106066	1.277659493	0.353503398
32	165.5683422	174.7995	0.040700273	0.030161979	2.023621904	0.019885323	0.326340236	1.349390036	0.432307414
33	166.0724454	174.739	18.69193478	14.43645157	2.018537294	0.025666884	0.344187166	1.29477349	0.372699732
34	168.0646757	280.5055	0.213330042	0.146895017	1.66322011	0.023269954	0.337645311	1.452261943	0.538301694
35	169.0959377	295.0025	0.162953412	0.124336285	1.108008375	0.028475525	0.350693443	1.310586146	0.390212186

36	172.038297	184.628	0.398748028	0.243842535	2.369371391	0.000379372	0.168878071	1.635268549	0.709527579
37	173.163827	235.915	0.097148559	0.053589609	1.174472626	0.02023058	0.327627334	1.812824536	0.858239293
38	177.0383652	43.249	0.288086382	0.2165325	1.684230228	0.039936513	0.395272587	1.330453316	0.411917889
39	181.06045	188.223	1.517977282	0.715966219	2.660370291	1.43258E-05	0.025695395	2.120180034	1.084186776
40	183.0389865	177.144	0.012584828	0.007901112	1.958052881	0.002479823	0.261305864	1.592791921	0.671557809
41	190.0520515	285.035	0.029735875	0.015735981	1.182993387	0.018133263	0.319239771	1.889674053	0.918137408
42	190.049875	184.4405	11.87706619	6.861755515	2.381856025	0.000412221	0.174691351	1.730907807	0.791528884
43	195.0767474	182.355	0.345824406	0.1936122	1.86855147	0.039475903	0.393440347	1.786170532	0.836869826
44	198.0869064	297.5545	3.124161282	2.092559141	1.38089974	0.027749935	0.349116505	1.492985895	0.578200536
45	201.1592596	50.4975	0.483895215	0.261723975	2.146697919	0.012123811	0.293210069	1.848876145	0.886648583
46	202.0700939	349.653	0.182162821	0.121064939	1.313355018	0.02176987	0.332975532	1.504670331	0.589447431
47	205.0074038	341.5725	0.152911955	0.109909721	2.29611194	0.006016635	0.27742674	1.391250501	0.476382207
48	206.1012946	281.771	0.075015547	0.057695617	1.348355956	0.048908135	0.426498089	1.300194895	0.378727895
49	208.0627537	285.428	0.327701557	0.162481218	1.060741265	0.008540786	0.282969887	2.016858077	1.012109567
50	213.0148947	276.492	0.043309336	0.029716965	1.772976352	0.025839436	0.344620036	1.457394326	0.543391279
51	215.1023944	293.221	1.915597383	1.441905601	1.926214748	0.012376659	0.293727694	1.328517888	0.409817653
52	215.1749067	49.0005	0.171608245	0.103833806	1.600342341	0.012803865	0.294559624	1.652720354	0.724842636
53	215.9957413	210.44	0.180720524	0.079598079	2.550222776	0.003608468	0.269642778	2.270413144	1.182954846
54	218.0471008	149.895	0.289966527	0.177437461	1.928916889	0.033687385	0.368324373	1.634190015	0.708575742
55	219.0688029	154.983	0.029487811	0.017887166	1.948774607	0.04067655	0.398163757	1.648545689	0.721193871
56	220.0957898	332.3755	0.28685032	0.187835598	1.769801056	0.004105906	0.271952409	1.527135022	0.610827624
57	221.0990069	332.3755	0.042308593	0.027045827	1.86189825	0.002900771	0.265108821	1.56432978	0.645544682
58	222.0963299	330.523	0.054571428	0.041842702	2.008032004	0.004082217	0.271854388	1.304204203	0.383169774
59	223.0180853	341.6225	0.061059472	0.045548473	2.132319746	0.012113374	0.293188278	1.340538292	0.42281243
60	223.073863	273.92	0.07097529	0.045079686	1.308345632	0.020643743	0.329123649	1.574440672	0.654839395
61	223.0815627	390.633	0.013594639	0.01117758	2.11373143	0.022395223	0.334982347	1.216241654	0.282429906
62	223.0957259	349.607	0.08172904	0.065563067	1.834746843	0.017016942	0.314150621	1.246571335	0.317965443
63	224.0369966	44.773	0.0833082	0.057952781	1.581258241	0.040083967	0.395853785	1.4375186	0.523580623
64	224.0583289	205.552	0.990319547	0.623098239	2.068944314	0.029856608	0.353518361	1.589347368	0.668434475
65	224.1270983	333.791	0.042309872	0.024544736	1.946213377	0.001094577	0.232250914	1.723785973	0.785580659
66	225.015139	236.348	0.023110794	0.015118477	1.721692671	0.006786572	0.278794902	1.528645644	0.612254013
67	228.0678801	346.598	0.054884137	0.039427661	2.107021762	0.024020623	0.339806635	1.392021125	0.477181105
68	230.2472658	137.0455	1.90305021	1.086710163	1.552735246	0.023243567	0.337567314	1.751203103	0.808346416
69	231.042421	81.4235	0.034793662	0.014972928	2.231139924	0.021982838	0.33366906	2.323771346	1.216468118
70	231.1452682	349.523	1.90210518	1.553828848	1.732386594	0.004381987	0.273021214	1.22414073	0.291769423
71	231.1806936	249.6025	0.131959011	0.09982567	1.598983471	0.047449873	0.421938128	1.321894568	0.402607115
72	232.1287922	292.649	3.726844038	2.716836772	1.680503076	0.018678355	0.321557109	1.371758538	0.456026555
73	234.0064078	210.506	0.431280139	0.18635955	2.574431688	0.00251836	0.261701735	2.314236859	1.21053653
74	234.0421604	139.344	0.07053155	0.048193435	1.989519281	0.032881941	0.364920062	1.463509499	0.54943211

75	237.0740526	331.776	0.028636868	0.021624457	1.730670681	0.015015996	0.303774301	1.324281467	0.40520979
76	237.1589931	210.822	0.039502835	0.025895006	1.46082989	0.045473587	0.415463525	1.525500137	0.609282309
77	238.1430078	226.831	0.303786453	0.177783761	1.738336324	0.021556609	0.332270292	1.708741287	0.772933982
78	239.0884155	347.962	0.232093822	0.172299213	1.780436919	0.022307675	0.334706747	1.34703936	0.429792006
79	239.2109926	145.548	0.070251381	0.201901576	1.191854423	0.043988964	0.410360966	0.347948652	-1.523053678
80	241.0285485	341.7985	0.294645309	0.216462775	2.015947984	0.011721999	0.29234563	1.361182351	0.444860351
81	245.1128585	399.342	0.900729113	0.529060769	2.432526068	7.99061E-05	0.065838088	1.702505961	0.76765985
82	245.185347	93.161	0.093583713	0.052609259	1.563155768	0.017850637	0.317996461	1.778844905	0.830940729
83	246.0397614	205.55	0.156956022	0.094896065	2.594475042	0.000624341	0.20197148	1.6539782	0.725940219
84	246.0786751	346.593	0.219680292	0.156080561	2.124530483	0.024179237	0.340249533	1.407480151	0.493114577
85	246.2419368	63.597	0.871557916	0.527912701	2.079460781	0.029539445	0.352889074	1.650950839	0.723297161
86	247.0426993	224.001	0.027185793	0.021523026	1.464008319	0.039999247	0.395520171	1.263102737	0.336971989
87	247.2450113	62.6315	0.156187119	0.08415028	2.175678534	0.012787769	0.294529204	1.856049899	0.892235497
88	248.166399	187.9615	0.075260805	0.048587503	1.466263581	0.04209419	0.403528033	1.548974535	0.631313426
89	248.9713731	341.5725	0.02508946	0.018347474	2.118144727	0.013752195	0.296235852	1.367461215	0.451499915
90	249.0895707	243.0335	0.225558412	0.168007764	1.760830114	0.042193469	0.403895406	1.342547552	0.424973188
91	251.0362249	279.9485	0.994805278	0.560266574	1.521307551	0.009703556	0.287034461	1.77559277	0.828300739
92	253.0937916	270.792	0.024116957	0.016572852	2.268940314	0.003333718	0.268093911	1.455208575	0.541225949
93	254.0916959	217.609	0.138462807	0.075343213	1.640412892	0.015682771	0.307393299	1.837760847	0.877949037
94	255.0422924	84.331	0.265288871	0.155514121	2.068343857	0.026260578	0.345657054	1.705882846	0.770518571
95	255.0789152	120.58	0.205968486	0.075515554	2.643677357	0.004701405	0.274109731	2.727497494	1.447577871
96	255.9876488	211.052	0.012581641	0.007050022	2.637478138	6.03372E-05	0.058634998	1.784624403	0.835620472
97	256.1032067	232.239	0.239935158	0.154482543	1.971017743	0.044723048	0.412910546	1.553153857	0.635200752
98	256.2626059	54.929	0.272372999	0.155734139	1.927738118	0.008419323	0.282488285	1.748961406	0.806498454
99	257.0736664	227.301	0.289251742	0.179799764	1.688856109	0.029557351	0.352924902	1.608743722	0.685934518
100	258.278334	133.355	0.015575243	0.008871496	1.544375506	0.031450597	0.359182058	1.755650097	0.812005343
101	260.2572896	57.8175	0.104564495	0.051597137	2.378444886	0.000256034	0.14059474	2.026556135	1.019030138
102	262.1285672	93.599	0.544600419	1.019210327	1.30503797	0.049256597	0.427561747	0.534335657	-0.904181802
103	263.0743374	118.215	0.095035265	0.071973402	1.967020797	0.045999898	0.41722227	1.320422025	0.400999108
104	263.1035857	231.9105	2.077019027	0.636566472	2.80983629	0.000439608	0.179127945	3.26284704	1.706131356
105	267.0970459	244.8285	0.159514668	0.08774783	2.066432247	0.01021092	0.288547152	1.81787594	0.862253747
106	267.9596437	144.311	0.031388875	0.017546813	2.273883058	0.002321319	0.259553939	1.788864724	0.839044293
107	269.1233516	311.785	0.051385481	0.040064605	1.948102602	0.013290959	0.295448099	1.282565511	0.359032518
108	272.1343392	367.834	0.106737282	0.076581286	2.168376555	0.019154771	0.323500063	1.393777609	0.479000383
109	272.2576151	66.859	0.245219782	0.094080345	1.934020774	0.011404932	0.291624436	2.606493219	1.382110107
110	272.2570358	54.135	0.106216381	0.059896583	1.860552009	0.013143368	0.295185285	1.773329572	0.826460685
111	273.0050774	279.887	0.076050822	0.052183874	1.10233653	0.017876358	0.31811083	1.457362519	0.543359792
112	273.0473248	227.3285	0.215927808	0.127833914	1.32092854	0.013619888	0.296014917	1.689127723	0.756278422
113	274.0217982	43.263	0.087004433	0.066027778	1.698423131	0.039482303	0.393465979	1.317694398	0.398015817

114	274.1748235	273.272	0.073563161	0.055132431	1.434932143	0.048364581	0.424819164	1.334299227	0.416082238
115	274.274255	54.617	150.8076712	76.62053811	2.011138574	0.019482858	0.324795756	1.968240826	0.976906754
116	277.1861651	292.725	0.114497795	0.078772536	1.699304504	0.016750039	0.312859689	1.453524289	0.53955518
117	279.1332908	280.872	0.316940621	0.239475779	2.036929998	0.031745185	0.360319707	1.323476731	0.40433283
118	285.0842626	231.973	0.27828909	0.119198963	2.312315243	0.003533223	0.269240835	2.334660326	1.223212665
119	288.1908674	273.597	0.556047156	0.376510618	1.446320631	0.034030606	0.369744604	1.476843226	0.562516685
120	289.0321806	127.016	0.041680326	0.030900778	1.7872822	0.007154964	0.279349194	1.348843918	0.431723416
121	289.0479358	255.305	0.012229599	0.008445045	2.047500573	0.035825145	0.377960585	1.448139021	0.534200108
122	291.1904431	348.46	0.026112908	0.019010873	2.130416722	0.013052184	0.295020184	1.373577488	0.4579383
123	294.099764	325.78	0.14290228	0.094339561	1.971898655	0.00127356	0.238934936	1.514765162	0.599094146
124	295.0915855	252.025	0.040288442	0.020821699	2.2608989	0.010976325	0.290589558	1.934925783	0.952278231
125	297.0016143	210.506	0.033207515	0.01703472	2.64622492	1.55929E-05	0.025695395	1.949401859	0.963031525
126	297.9858958	255.6765	0.005974561	0.004336806	1.784711313	0.01343268	0.295695452	1.377640853	0.462199831
127	302.0860039	347.2775	0.216804922	0.158459545	1.459288759	0.0251039	0.342741388	1.368203612	0.452282944
128	302.2077406	265.4265	2.012771468	1.403723397	1.189325047	0.02939263	0.352593952	1.433880402	0.519924696
129	302.3044126	52.5375	0.510340432	0.208224683	1.983361067	0.014723508	0.302112406	2.45091227	1.293318844
130	305.0848948	145.7055	0.557768318	0.452572426	1.393386619	0.027590985	0.348761944	1.232439907	0.301517304
131	307.0584232	418.926	0.079001417	0.042546422	2.282500167	0.023569273	0.338520288	1.856828701	0.892840727
132	307.078748	311.965	0.073084473	0.055128384	2.202220689	0.0139731	0.2976232	1.325714046	0.406769622
133	309.0951715	304.936	0.404531601	0.266679243	1.607699013	0.014241828	0.29926956	1.516921962	0.601146868
134	310.0555656	340.929	0.056681164	0.02899518	2.320375651	0.00112082	0.233338413	1.95484779	0.96705628
135	310.1749071	211.1885	0.103387598	0.068105948	2.042445114	0.024073382	0.339954473	1.518040656	0.60221043
136	312.1106136	325.7605	0.272138674	0.178682117	1.768990162	0.00332644	0.268049663	1.523032516	0.606946743
137	314.2171984	432.845	0.010911996	0.016176286	1.299660849	0.027309553	0.348125858	0.674567469	-0.567965348
138	316.1017612	288.8485	0.367463554	0.286430579	1.486686417	0.031250155	0.358399913	1.282906156	0.359415642
139	318.2993859	56.6025	2.895899568	1.647188245	2.111861074	0.015363687	0.30568995	1.758086593	0.814006131
140	322.9408707	344.393	0.014870835	0.010860688	2.068991062	0.026907597	0.34719847	1.369235001	0.453370077
141	325.0994269	345.2705	0.081765898	0.056383254	1.763565194	0.017731452	0.317463248	1.450180553	0.536232533
142	326.0367219	177.292	0.180190916	0.116694724	2.032505002	0.010969569	0.290572658	1.544122209	0.626786939
143	327.051376	319.0435	0.073481858	0.046398811	1.24248422	0.038735191	0.390439664	1.583701316	0.663300271
144	327.0862582	282.402	0.017326578	0.010736797	1.554361266	0.00600375	0.277400987	1.613756766	0.690423144
145	329.0477966	37.027	0.074375838	0.023366045	2.131617847	0.000745932	0.21248254	3.183073494	1.670420468
146	330.1904083	350.183	0.030949706	0.022580573	1.608787089	0.026641589	0.346572159	1.370634235	0.454843628
147	331.1487221	239.468	0.030823647	0.019255927	1.871154238	0.021254594	0.331252625	1.600735588	0.678735021
148	339.0636035	175.51	0.16062625	0.12045023	1.572554384	0.017383211	0.315874074	1.333548716	0.415270529
149	341.1695223	332.604	0.15590064	0.089730892	1.70037209	0.023737072	0.339003084	1.737424391	0.796950196
150	344.0968433	280.569	0.17030791	0.111321382	1.601710143	0.024063098	0.339925696	1.529875995	0.613414719
151	344.2059728	240.2515	0.255912184	0.174874054	1.377590638	0.021445075	0.331897082	1.463408539	0.549332582
152	345.0346224	312.1755	0.080007395	0.061628346	2.245834895	0.010264414	0.288698803	1.298223943	0.376539269

153	345.1757926	350.7625	0.179237765	0.147360485	1.552888378	0.04405324	0.410586324	1.216321759	0.282524921
154	346.1597927	369.99	0.07826038	0.064005663	1.527673264	0.045344062	0.41502675	1.222710238	0.29008255
155	346.2328221	265.173	0.032673371	0.023495268	1.61438499	0.027537928	0.348642845	1.390636255	0.475745107
156	347.0533674	244.8285	0.061303553	0.033476275	2.409102099	0.005010597	0.275038016	1.83125374	0.872831706
157	347.0764896	29.1045	0.039427261	0.016546281	1.5349974	0.038192837	0.388198986	2.38284731	1.252686508
158	350.0863292	235.2435	0.275860384	0.06519908	2.262801898	0.011180539	0.291091613	4.231047182	2.081014774
159	351.0681818	327.316	0.320336614	0.249028154	1.569785942	0.025076891	0.342670707	1.286346983	0.363279852
160	355.0731713	177.095	0.032648814	0.019060608	1.554335424	0.020989873	0.330341858	1.712894666	0.776436436
161	355.0841656	335.895	0.028966182	0.017226958	1.674395288	0.007854723	0.280263682	1.681444974	0.749701567
162	357.9790758	341.006	0.046966212	0.032081299	2.249831481	0.006060536	0.277513695	1.463974747	0.549890668
163	359.0667495	325.751	0.014843976	0.011290435	1.837744926	0.009105173	0.285057933	1.314739122	0.394776561
164	365.0069799	318.716	0.04989151	0.036302449	1.961358638	0.01811281	0.319150776	1.374329074	0.458727489
165	367.1425205	346.773	0.736185621	0.5108092	2.243791676	0.013520795	0.295846833	1.44121449	0.527285062
166	368.135932	297.757	0.232838256	0.107494675	1.809529714	0.025722934	0.344328292	2.166044568	1.115062928
167	368.9741496	341.594	0.038500501	0.029208785	2.106781488	0.011983137	0.29291345	1.318113753	0.398474881
168	369.0656485	256.6545	0.057168293	0.04431578	1.923414951	0.037409421	0.384895272	1.290021136	0.367394703
169	371.0044035	43.154	0.055635983	0.042025772	1.633369804	0.045484635	0.415500708	1.323853904	0.40474392
170	376.0499639	279.945	0.341546341	0.186182925	1.681517747	0.010340796	0.28891289	1.83446651	0.875360567
171	376.2585755	33.7385	0.235203983	0.120556418	1.295988148	0.008152827	0.281387543	1.950986827	0.964204037
172	377.063275	237.182	0.021896512	0.01393113	1.830696958	0.04662124	0.419265996	1.571768544	0.652388785
173	377.0996873	417.5175	0.017045241	0.010785373	2.171539154	0.028872613	0.351528615	1.580403504	0.66029295
174	379.9604392	341.077	0.027246425	0.01870966	2.142300854	0.005227568	0.275627107	1.45627578	0.54228359
175	382.0755076	361.3185	0.141438241	0.09550402	2.021227913	0.03660507	0.381417966	1.480966365	0.566538875
176	382.9900495	312.2485	0.015013808	0.011598612	1.819986867	0.027137392	0.34773141	1.294448631	0.372337714
177	385.9918988	37.061	0.009738028	0.006133925	1.579599161	0.005331898	0.275894127	1.587568701	0.666819025
178	387.1802286	37.0385	4.472069446	2.786065383	1.388057657	0.009179676	0.285316512	1.605155957	0.682713476
179	388.0216099	325.46	0.025764245	0.017343326	2.054524378	0.022146291	0.334194207	1.4855423	0.570989685
180	393.2847872	33.718	0.28156351	0.151050068	1.259168265	0.008833207	0.284081237	1.864040939	0.898433545
181	394.0757902	124.86	0.371202934	0.188065378	1.393907248	0.040317031	0.39676719	1.973797289	0.980973831
182	397.1997039	36.003	0.049164062	0.027429165	1.72261457	0.002822619	0.264480984	1.792400985	0.841893424
183	398.0195088	279.872	0.085435818	0.054443377	1.339097465	0.004859508	0.274598379	1.56926008	0.650084476
184	398.05495	382.95	0.021820277	0.009734413	1.64956876	0.04672961	0.419618899	2.241560629	1.164503522
185	399.0361578	275.257	0.018457128	0.011195958	1.425001677	0.032217967	0.36211658	1.648552844	0.721200133
186	402.0767716	290.602	0.041989791	0.023575905	2.018315844	0.004603841	0.273792378	1.781046869	0.832725482
187	403.0782849	29.3165	0.128990993	0.069068074	1.844067208	0.025079843	0.342678439	1.86759215	0.901179429
188	404.2057511	37.1185	0.803788918	0.491639064	1.406111729	0.007707021	0.280084011	1.634916704	0.709217135
189	409.3271098	137.023	0.022055255	0.013587262	1.957061902	0.012192396	0.293352414	1.623230246	0.698867652
190	410.1296741	359.9485	0.092672812	0.045079534	2.36534667	0.00454735	0.273602753	2.055762448	1.039673565
191	410.1902397	414.645	0.074530444	0.036337648	2.320456651	0.014360051	0.299979915	2.051053038	1.036364799

192	410.2379496	298.369	0.028810553	0.015131389	2.352987597	0.005402591	0.276069477	1.904025707	0.929052957
193	412.1618678	329.2205	0.06316159	0.030276169	1.996294301	0.022979913	0.336780179	2.086181696	1.060864815
194	413.0527533	303.1495	0.030479923	0.012845268	1.182592829	0.042328907	0.404394876	2.372852212	1.246622248
195	413.1220864	299.581	0.237624699	0.119842562	1.426913613	0.010095639	0.288215434	1.982807232	0.987544426
196	413.9364356	36.326	0.006685205	0.004103901	1.41300291	0.008840304	0.284107399	1.628988112	0.703976075
197	414.0399227	340.8725	0.026391801	0.015428828	1.977486481	0.026558245	0.346373818	1.710551212	0.774461297
198	415.2110272	36.225	3.297760051	2.02882672	1.187761046	0.025465207	0.343675204	1.625451803	0.700840779
199	416.9349522	341.505	0.019241358	0.014230701	1.928253365	0.028329429	0.350381297	1.352101884	0.435203866
200	420.1415608	325.2415	0.036063601	0.027489613	1.185948793	0.039656189	0.394160537	1.311899203	0.391656878
201	428.0174637	38.3795	0.005717496	0.010236616	1.052644664	0.04216222	0.403779885	0.55853377	-0.840283583
202	432.2374438	37.0305	2.175009855	1.68388357	1.196137777	0.03497206	0.374078472	1.291662852	0.369229549
203	441.1925644	273.857	0.02966124	0.018050124	1.477619957	0.005499047	0.276301802	1.643270698	0.716570157
204	444.1349404	257.476	0.07825034	0.157006644	1.073298296	0.029794257	0.353395532	0.498388719	-1.004656682
205	449.3470932	34.024	0.08465226	0.059571316	1.137615035	0.038673305	0.390185873	1.421023839	0.506930758
206	450.1255105	395.9555	0.108381127	0.057312882	1.771535034	0.036801643	0.382275928	1.89104305	0.919182207
207	456.0999708	29.3305	0.041770893	0.02930954	1.881633917	0.0226739	0.3358484	1.425163689	0.511127632
208	456.1691686	25.0635	0.026567767	0.018412647	2.030680216	0.010955917	0.290538452	1.442908599	0.528979915
209	463.2369376	370.5885	0.026247606	0.019754559	1.762029495	0.013607984	0.295994845	1.328686005	0.410000208
210	463.2492373	292.462	0.012554745	0.008539139	1.093957001	0.031818472	0.360600559	1.470258791	0.556070116
211	463.942132	341.006	0.016319646	0.011845068	1.909317245	0.041438528	0.401074857	1.377758719	0.462323257
212	464.0444218	307.561	0.017576809	0.013608329	2.191200157	0.008307271	0.282033026	1.291621396	0.369183244
213	466.1627036	32.4815	0.020367545	0.014966789	1.663454431	0.014141673	0.298661161	1.360849382	0.444507399
214	470.1299091	279.275	0.016931791	0.008896284	1.878926846	0.006151799	0.277690658	1.903243088	0.928459839
215	476.0778627	125.588	0.037544548	0.023327025	1.808309711	0.006544878	0.278398699	1.609487182	0.686601087
216	477.3416512	33.763	0.04946768	0.033896949	1.412994754	0.025922635	0.344827078	1.459354928	0.545330802
217	478.0591951	315.6885	0.007738518	0.006165461	1.928028789	0.042700546	0.405755368	1.255140092	0.327848399
218	479.1309567	278.58	0.015239514	0.010385442	1.560987104	0.011963111	0.292870707	1.467391979	0.553254305
219	489.0507636	317.822	0.015870955	0.01160181	1.352623557	0.025669824	0.344194579	1.367972308	0.452039026
220	489.1003278	28.24	0.046519714	0.035472574	1.926192722	0.033351024	0.366915035	1.311427654	0.391138222
221	491.3057823	49.228	0.055583747	0.029458031	2.129543956	0.003576532	0.269474105	1.886879204	0.916002066
222	501.0643071	279.933	0.796666714	0.409302875	1.392720345	0.012423534	0.293821537	1.946399021	0.9608075
223	501.9999165	307.854	0.004800093	0.003542875	1.955255561	0.033192942	0.366246603	1.354858088	0.438141747
224	502.2908863	180.416	0.07310995	0.043187964	1.849196808	0.044385407	0.411744426	1.692831602	0.759438466
225	513.3204752	49.433	0.051896441	0.023888785	2.110593063	0.002399176	0.260440462	2.172418625	1.119302137
226	514.3733671	48.296	0.010781024	0.005304337	2.25881035	0.001011624	0.228520985	2.032492408	1.023249963
227	518.089821	279.872	0.04702122	0.026603856	1.426458969	0.018344596	0.320150552	1.767458869	0.821676642
228	520.3370592	177.073	0.042408195	0.02031375	1.649356706	0.012457483	0.293889098	2.087659548	1.061886459
229	523.0437015	279.9205	0.058790472	0.040924333	1.164415344	0.017561038	0.316691457	1.43656519	0.522623463
230	524.3191857	38.838	0.027902305	0.011882524	1.958131929	0.007870421	0.280282394	2.34818005	1.231543033

231	524.3683158	174.2025	0.019352238	0.011954037	1.81953723	0.023106963	0.337161268	1.618887209	0.695002474
232	529.1388749	231.676	0.027523592	0.012459714	2.694829958	0.001433427	0.243718886	2.209006709	1.143397801
233	533.2259001	37.859	0.023880309	0.017052909	1.184029294	0.039853878	0.394945751	1.400365709	0.48580364
234	535.3322203	49.749	0.059839191	0.032374956	1.994220551	0.009291352	0.285697194	1.848317297	0.886212442
235	543.3380913	48.267	0.055353223	0.020771288	2.056086851	0.006307518	0.277981267	2.664891198	1.414076632
236	544.3369482	173.2595	0.029046878	0.014241359	2.042720665	0.008806627	0.283982918	2.03961417	1.028296266
237	547.1761488	231.7975	0.01045514	0.004506995	2.651273236	0.00384438	0.270807722	2.319758777	1.213974792
238	548.1699748	196.182	0.036202176	0.024802756	1.699471768	0.017859665	0.318036632	1.459602953	0.545575975
239	552.2162603	400.057	0.041151448	0.031140125	1.833790404	0.007277977	0.279522235	1.321492688	0.402168443
240	558.3989258	49.579	0.033077648	0.018782608	1.620205632	0.039938536	0.395280579	1.761078503	0.816459221
241	561.2132181	258.5315	0.019458934	0.039903674	1.005516379	0.026469722	0.346162035	0.487647681	-1.036088898
242	563.2194997	209.766	0.231797267	0.872134633	1.664233589	0.015791683	0.307963146	0.265781519	-1.911687304
243	565.351708	49.037	0.054915869	0.02093057	1.999036278	0.005459405	0.276207268	2.623715816	1.391611465
244	567.0524956	122.571	0.004018318	0.002501237	1.469176494	0.033581051	0.367880722	1.606532067	0.683949778
245	568.1829742	415.966	0.304476085	0.223461133	1.840560723	0.022089707	0.334013103	1.362546054	0.446304994
246	569.1194115	456.871	0.018503765	0.014092441	2.074374083	0.042812665	0.40616295	1.313027635	0.39289728
247	582.1512483	32.198	0.069742343	0.041080247	1.958044966	0.033862983	0.369053217	1.697709948	0.763589997
248	584.3556554	35.6685	0.017314176	0.005392208	1.952716665	0.015447285	0.306141176	3.210962037	1.683005608
249	587.2286638	257.913	0.017212043	0.037558798	1.017903102	0.035271534	0.37545349	0.458269279	-1.12573252
250	587.3648596	49.2345	0.044584907	0.018052039	1.91475815	0.004608623	0.273808231	2.469798922	1.30439359
251	588.1400722	374.581	0.024724853	0.015309676	2.241995747	0.016787559	0.313042995	1.614982049	0.691518129
252	591.2458047	137.311	0.019365223	0.012766288	2.029476197	0.036987389	0.383081759	1.516903178	0.601129003
253	598.2822121	332.4835	0.008796969	0.006800752	1.643744023	0.029413275	0.352635601	1.293528912	0.371312301
254	599.2422529	275.2435	0.009902443	0.005173584	1.854828101	0.030806561	0.356645258	1.914039389	0.936620519
255	603.2450965	380.9255	0.013995945	0.009966893	2.271807643	0.007059543	0.279210966	1.404243445	0.489793068
256	608.3106367	370.6595	0.026634527	0.021718924	1.933224174	0.049112826	0.427124083	1.2263281	0.294345018
257	609.3785529	49.437	0.035248131	0.014976949	1.739281134	0.009414774	0.286108549	2.353492072	1.234802993
258	612.3708662	42.321	0.055324655	0.021136635	1.794640761	0.007371298	0.279649794	2.617476995	1.388176857
259	617.3811667	48.948	0.01905223	0.007396183	1.909297592	0.011551348	0.291961947	2.575954267	1.36510698
260	620.0457292	210.746	0.009624551	0.002027849	2.471201753	0.020152634	0.327339734	4.746186094	2.246768669
261	626.0780637	279.921	0.543072494	0.26151041	1.361551593	0.014775795	0.302412976	2.076676389	1.054276417
262	626.5812414	279.3205	0.006145354	0.002788783	1.30707113	0.023878474	0.339405711	2.203597157	1.139860507
263	628.3811115	37.035	0.027715436	0.007585977	1.835988356	0.013668692	0.296096872	3.653508965	1.869282747
264	630.1973432	352.7455	0.069804745	0.099359121	1.486034114	0.031733657	0.360275447	0.702549943	-0.509327307
265	631.3906356	49.762	0.028612827	0.012082271	1.973975694	0.006331723	0.278025211	2.368166409	1.243770462
266	635.1616498	195.5305	0.011385965	0.008179206	1.490937063	0.045814699	0.416606318	1.392062322	0.477223801
267	635.1982979	95.8	0.201169644	0.156481487	1.274824645	0.048486101	0.425196625	1.285581109	0.362420634
268	640.38721	48.322	0.011095072	0.006613605	1.775449443	0.008519521	0.282886445	1.677613258	0.746410168
269	643.104469	279.817	0.011652822	0.006403901	1.568070952	0.021123592	0.330804143	1.819644364	0.863656514

270	648.0492617	279.952	0.024944913	0.018272432	1.132879006	0.028544011	0.350838858	1.365166538	0.449076958
271	653.1057809	380.1325	0.007758397	0.0026888	2.375672409	0.018095413	0.319074958	2.885450029	1.528796346
272	654.1360328	446.7795	0.011708492	0.00925233	1.861988799	0.044588188	0.412446102	1.26546409	0.339666568
273	656.088355	279.292	0.01279679	0.008741169	1.335937501	0.04876282	0.426051616	1.463967912	0.549883933
274	656.3971871	44.2065	0.069999643	0.027777262	1.911683674	0.006975725	0.279086545	2.520033965	1.333443178
275	660.4862449	46.2345	0.007960073	0.004634571	1.611451354	0.027652383	0.348899299	1.717542481	0.780345783
276	661.3990292	48.0915	0.011146384	0.005203416	1.950610796	0.006197043	0.277776535	2.142128293	1.099044886
277	668.0991554	278.6295	0.00588937	0.00382267	1.856571331	0.035085268	0.374599828	1.540643151	0.623532739
278	672.4068755	38.373	0.043937426	0.010941186	1.954360334	0.014398378	0.300208419	4.015782753	2.005681224
279	678.4749915	42.303	0.03067241	0.02010336	1.234292477	0.042634946	0.405516283	1.525735519	0.609504897
280	679.3374085	402.122	0.017471472	0.012675467	1.8551288	0.047418672	0.421838599	1.378369051	0.462962214
281	684.412051	48.4895	0.00945228	0.005905444	1.823102086	0.007687402	0.280059643	1.600604537	0.678616904
282	700.423865	45.577	0.078466068	0.029911374	2.016240238	0.005691043	0.276741875	2.623285323	1.391374732
283	701.2625813	324.5375	0.027878993	0.01801068	2.01619949	0.002778281	0.26411049	1.547914467	0.630325755
284	704.5102399	46.8355	0.003994968	0.002586985	1.894770227	0.006197603	0.277777592	1.544256711	0.626912601
285	704.5747102	162.829	0.041222431	0.027845744	1.910670261	0.027130051	0.347714498	1.480385354	0.565972768
286	705.4342434	49.005	0.006730059	0.002738426	1.945618962	0.017888388	0.318164237	2.457637386	1.297272068
287	716.4333462	39.6035	0.047797148	0.012677372	1.828281358	0.015461515	0.30621763	3.770272663	1.914668862
288	717.0292914	210.44	0.010094343	0.002249185	2.420095219	0.022362424	0.334879296	4.488000253	2.166072757
289	718.5274592	46.831	0.004036603	0.002584603	1.804109001	0.00482731	0.274501324	1.56178851	0.643199104
290	719.1475572	28.964	0.017496452	0.014215387	1.818412994	0.034941021	0.373935192	1.230810805	0.299609014
291	722.3438645	214.795	0.002438599	0.009498305	1.737042529	0.007621696	0.279977154	0.256740441	-1.96161753
292	722.5002974	44.2355	0.027725002	0.017386531	1.405686963	0.029736355	0.353281084	1.594625272	0.673217438
293	723.9751304	210.468	0.002601111	0.000670035	2.257996081	0.00270303	0.26345641	3.882049214	1.956818408
294	724.0938847	81.515	0.004165217	0.002631459	1.834263489	0.029792653	0.353392367	1.582854862	0.662528975
295	724.5241617	125.8175	0.134307202	0.067790702	1.924346012	0.03001653	0.353831456	1.981203895	0.986377362
296	732.4451412	35.205	0.009257682	0.002475972	1.737449996	0.026199347	0.345507966	3.739009429	1.902656109
297	740.0935982	43.161	0.040441947	0.029414733	1.69018182	0.030938809	0.357171827	1.374887446	0.459313519
298	741.2200858	427.745	0.074383743	0.031436506	2.432451201	0.007075044	0.279233666	2.366158051	1.242546444
299	744.4500297	46.8445	0.078327299	0.030392449	2.012024824	0.005326858	0.275881457	2.577196051	1.365802289
300	751.0920874	279.908	0.177723774	0.092261056	1.377631138	0.016775203	0.312982698	1.926314104	0.945842968
301	752.5109134	46.8245	0.005625273	0.004417453	2.023249524	0.013191997	0.295272478	1.273420177	0.348708529
302	758.245302	427.308	0.016515588	0.008454215	2.167387888	0.019908942	0.326429382	1.953533078	0.966085684
303	760.4591447	41.323	0.064984972	0.01787421	1.789538824	0.011647412	0.292179193	3.63568354	1.862226629
304	760.5752346	44.773	0.012972765	0.008115307	1.73650532	0.006981269	0.279094863	1.598555006	0.676768388
305	763.2010494	427.587	0.012021471	0.005914511	2.438006017	0.005776689	0.276929174	2.032538215	1.023282478
306	766.5275247	45.778	0.023141599	0.01466621	1.485454911	0.015562202	0.306755668	1.577885413	0.65799244
307	766.5711674	120.148	0.041381751	0.018428259	2.040122287	0.02004996	0.326958257	2.245559485	1.16707494
308	772.4682605	49.162	0.008265373	0.004722619	1.893080761	0.007826818	0.280230239	1.750167414	0.807492931

309	773.0732023	279.945	0.075025236	0.049597717	1.018150781	0.013714495	0.296173299	1.512675196	0.597102243
310	776.4696335	36.923	0.013696238	0.003042892	1.796660007	0.016738295	0.312802191	4.501059278	2.170264565
311	776.5698239	46.725	0.013425321	0.007958039	1.768919838	0.012185741	0.293338666	1.687013745	0.754471728
312	781.1035894	279.251	0.006076854	0.004049224	1.658674063	0.024372185	0.340782054	1.500745473	0.585679316
313	781.2063731	217.4085	0.007388469	0.018688611	1.764365563	0.009724234	0.287098873	0.39534605	-1.338812084
314	781.2464641	347.197	0.006955718	0.005520836	1.388009167	0.032657808	0.363954466	1.259903121	0.333312803
315	784.233302	391.186	0.016218872	0.008467539	2.074111966	0.038933778	0.391250819	1.915417554	0.937658929
316	788.4763918	47.556	0.073684963	0.028869558	2.070056863	0.003216092	0.267356215	2.552341206	1.351821207
317	796.2181948	210.779	0.013479002	0.045490376	1.042134348	0.02085935	0.329886175	0.296304486	-1.754847629
318	801.2603726	391.2305	0.006712812	0.003893648	1.954838589	0.032254478	0.362253889	1.724041659	0.785794635
319	804.4859931	42.7705	0.075546673	0.022677576	1.914907812	0.009164879	0.285265452	3.331338045	1.736101758
320	805.2108199	390.99	0.010978221	0.006080486	1.975956278	0.046208864	0.417913499	1.805484174	0.852385775
321	810.5538735	46.8305	0.019028156	0.01043193	1.802693383	0.003078645	0.266428591	1.824030142	0.86712957
322	811.2177839	222.322	0.004193518	0.009708696	1.233279465	0.025477099	0.343705573	0.431934244	-1.211116395
323	812.5390157	176.525	0.029773807	0.016717191	1.740902272	0.045219818	0.4146063	1.781029293	0.832711245
324	816.492742	49.5675	0.006135138	0.003403134	2.040420792	0.005974647	0.277342431	1.802790853	0.850232035
325	820.4964485	38.024	0.018049515	0.00420614	1.745738194	0.018861616	0.322313329	4.291230472	2.101391387
326	824.4979019	32.8535	0.008257342	0.004589166	1.691257997	0.01135372	0.291504518	1.799312332	0.847445637
327	832.5006299	48.188	0.060202013	0.024174833	2.059692617	0.005805255	0.27699047	2.490276298	1.316305819
328	835.3519943	397.388	0.024899077	0.018786471	1.883797228	0.042544594	0.405186244	1.32537276	0.406398174
329	837.0778473	43.249	0.4980533	0.361653828	1.467302596	0.037950976	0.387187607	1.377154787	0.461690722
330	838.2022536	430.298	0.006048804	0.003023992	2.265052702	0.010339444	0.288909125	2.000271371	1.00019574
331	839.2475486	213.942	0.009739952	0.036683985	1.268297929	0.011997769	0.292944598	0.265509636	-1.913163876
332	846.2743494	196.033	0.010904307	0.006588131	1.242431496	0.007042672	0.279186152	1.655144354	0.726957048
333	848.5126597	44.401	0.077359355	0.023415147	1.889677856	0.009572446	0.28662027	3.303816726	1.724133658
334	854.5803689	47.545	0.013371865	0.007363549	1.773313938	0.002484996	0.261359649	1.815953855	0.860727543
335	864.5214291	39.218	0.016853831	0.003940188	1.777498464	0.021646047	0.332567385	4.277418404	2.096740334
336	865.1070214	43.021	0.015965927	0.011844595	1.672083301	0.044714984	0.412882823	1.347950399	0.43076741
337	876.1061653	279.872	0.138019654	0.064763597	1.450373928	0.015725876	0.30761952	2.131130139	1.091618695
338	876.5287679	48.318	0.043687264	0.018648184	1.998554387	0.004763333	0.274304789	2.342708785	1.228177628
339	892.5382494	45.783	0.069578531	0.020316473	2.072254992	0.007573188	0.279915369	3.424734695	1.775992231
340	893.1321173	279.8595	0.023538923	0.010673873	1.683331081	0.010631047	0.289701091	2.205284023	1.140964476
341	898.0775868	279.955	0.011766598	0.007984217	1.209929433	0.015634215	0.307137374	1.473732297	0.559474483
342	908.5478775	40.886	0.01875934	0.004459846	1.889089592	0.022846548	0.33637653	4.206275228	2.072543253
343	920.5541402	48.487	0.028630874	0.012867809	2.115138872	0.003295211	0.267857764	2.224999974	1.153805319
344	936.5641031	46.826	0.055540882	0.016961129	2.091130047	0.004533531	0.273555689	3.274598205	1.711317898
345	937.4348625	394.596	0.01984058	0.016041773	1.901595931	0.04552142	0.415624426	1.236807191	0.306620613
346	939.3355577	214.019	0.002176523	0.006070903	1.222901578	0.01941814	0.324542825	0.358517134	-1.479886025
347	952.5743559	42.224	0.022784303	0.006739163	1.854663996	0.010812209	0.290173654	3.380880096	1.757398852

348	954.9729421	210.44	0.00601071	0.001076934	2.066649998	0.007910336	0.280329651	5.581315369	2.480605167
349	964.5809531	48.713	0.018922237	0.009371658	1.969704826	0.004193277	0.272304948	2.019091786	1.013706496
350	969.1677136	279.2955	0.002970405	0.001997319	2.186306853	0.013229873	0.295339979	1.487196347	0.572595131
351	980.590961	47.506	0.039843395	0.0124171	2.046490851	0.006415314	0.278174514	3.208751997	1.682012289
352	996.6006931	43.9395	0.018059141	0.005400076	1.757999408	0.013509752	0.295827961	3.344238213	1.741677615
353	1001.120997	279.8405	0.023799583	0.012474887	1.764917265	0.025751069	0.344398943	1.907799513	0.931909569
354	1003.103166	42.4875	0.039439739	0.023093034	1.759985111	0.047305479	0.42147681	1.707863021	0.772192268
355	1008.608571	49.0675	0.012621787	0.006646093	1.956114141	0.011539696	0.291935374	1.899128975	0.925337886
356	1018.147849	279.872	0.095642646	0.045594352	1.661509306	0.017005046	0.314093721	2.097686248	1.068798909
357	1018.65087	279.833	0.003514275	0.001665107	1.909203473	0.049655308	0.428766849	2.110539721	1.077611982
358	1023.102069	279.921	0.015446075	0.010161361	1.257964329	0.018662038	0.321489234	1.520079395	0.604146679
359	1040.627468	45.3805	0.013560928	0.003619332	1.920565076	0.007058768	0.279209829	3.746804119	1.905660556
360	1052.632802	49.483	0.008621404	0.004233215	2.15267504	0.001787224	0.251665172	2.036608952	1.026168996
361	1077.263045	24.709	0.007713522	0.005440829	1.80321997	0.034150343	0.370235888	1.417710795	0.503563261
362	1084.65126	46.826	0.00934992	0.002649326	1.999588323	0.00715916	0.279355191	3.529169019	1.819328526
363	1094.083119	43.3725	0.005895688	0.004043802	1.805359119	0.017896107	0.318198477	1.457956621	0.543947796
364	1096.657501	50.542	0.004858786	0.002596936	1.976057895	0.004936822	0.274826535	1.870968665	0.903785397
365	1110.092245	43.256	0.223989079	0.161044199	1.402993325	0.048339157	0.424740038	1.390854689	0.475971701
366	1111.206157	279.952	0.001996993	0.001264549	1.908486262	0.022843397	0.33636695	1.57921347	0.659206201
367	1126.136687	279.5675	0.006716336	0.003641331	1.88453061	0.037832935	0.386691242	1.844472592	0.883208351
368	1138.123243	279.393	0.003702652	0.002029133	1.645404857	0.027890252	0.349426733	1.824745693	0.867695416
369	1143.161664	279.8795	0.090903571	0.043224316	1.710007226	0.018932843	0.322604237	2.103065564	1.072493827
370	1194.882913	29.704	0.013898573	0.005252555	1.331185694	0.042251501	0.40410966	2.646059631	1.403845574

Note: The metabolites were selected by the condition of VIP > 1. $p < 0.05$.

Table S3. The differentially expressed urine metabolites in negative mode by LC-MS/MS analysis.

No.	mz	rt	MEAN LF	MEAN CONTROL	VIP	*p*-VALUE	Q-VALUE	FOLD CHANGE	LOG_FOLDCHANGE
1	74.02498377	334.507	0.232576711	0.166144931	1.288624265	0.021341193	0.32635417	1.399842356	0.485264367
2	74.02475713	260.476	0.616858789	0.467240329	1.2113933	0.033040393	0.368705565	1.32021735	0.400775463
3	75.00869301	301.945	0.369738422	0.287611343	1.85726956	0.012554669	0.300777279	1.285548819	0.362384398
4	79.95753583	279.894	2.816811872	1.797876019	1.53551784	0.004339169	0.245794189	1.566744226	0.647769676
5	84.04523353	282.854	0.100795066	0.076873627	1.448168893	0.044809296	0.403183293	1.311178747	0.390864375
6	98.02449893	260.476	0.28181478	0.205616185	1.258727026	0.019485275	0.322126924	1.370586563	0.454793448
7	102.0555842	339.568	0.288381849	0.217184172	1.709489429	0.008926516	0.286302996	1.327821663	0.409061394
8	104.0350585	282.8045	0.097642043	0.06994914	1.435269222	0.021177007	0.325998973	1.39590054	0.481196151
9	108.0120785	326.501	0.05676887	0.026103344	1.141504924	0.016990647	0.316158221	2.174773867	1.120865397
10	108.0451522	212.003	0.683922945	0.361475979	1.50831491	0.001988257	0.185777906	1.892028752	0.919934012
11	109.0653203	331.666	0.101054385	0.077125216	1.184064447	0.047166629	0.408810541	1.310263883	0.389857395
12	112.0402678	333.561	0.17903497	0.075349098	2.42510971	1.09924E-05	0.015296932	2.37607318	1.24857927
13	115.0032219	42.9565	4.994746904	4.150340385	1.18807675	0.030605804	0.361645562	1.203454763	0.267181913
14	117.0188171	83.2975	2.342462028	1.723415061	2.031685235	0.006350834	0.26927807	1.35919784	0.442755465
15	121.078997	24.875	3.145096258	2.140630122	2.062237668	0.000729527	0.128656376	1.469238531	0.555068637
16	122.0243687	410.5385	0.302059466	0.187899942	1.891071045	0.00015359	0.052013159	1.60755487	0.684867981
17	124.0079304	280.0925	23.98974707	15.48446686	1.224880079	0.01165463	0.297146181	1.549278208	0.631596236
18	128.0358512	282.122	7.67150271	5.724885742	1.291086374	0.034549121	0.372699796	1.340027217	0.422262304
19	129.0551472	51.899	3.494520579	2.394625295	1.108454401	0.009702834	0.289919095	1.459318327	0.545294618
20	130.9982859	295.9095	0.094318432	0.060934653	1.971244512	0.007712621	0.279446526	1.547861959	0.630276816
21	133.0501899	68.929	0.431544539	0.242056297	1.858698571	0.005911987	0.265220782	1.782827156	0.834166842
22	134.061155	189.011	35.9383991	27.9984856	1.150801595	0.046043941	0.406183003	1.283583677	0.360177349
23	135.0302094	301.761	5.834606139	4.326673669	2.015206977	0.010152877	0.291797565	1.348520038	0.431376959
24	141.0918525	323.6015	0.12960957	0.099882992	1.549841425	0.028324624	0.354231979	1.297614012	0.375861303
25	142.0504263	211.437	0.307421034	0.22992552	1.582110327	0.025519713	0.34386065	1.337046161	0.419049274
26	145.0614925	293.805	1.085821635	0.820414554	1.109358179	0.047068911	0.408585498	1.323503624	0.404362145
27	152.0350727	212.003	2.536513352	1.355262047	1.533260741	0.001467468	0.170232174	1.87160362	0.904274924
28	156.0661044	192.252	0.801085557	0.53647019	1.786254004	0.025901638	0.345346759	1.493252695	0.578458325
29	157.0137326	46.851	2.719821341	1.23921643	2.220979434	0.001904207	0.183691561	2.194791221	1.13408371
30	157.0136076	196.915	0.363956971	0.287231784	1.341371745	0.048089674	0.410902972	1.267119418	0.341552495
31	158.0534092	357.27	0.013190414	0.01034349	1.375868183	0.046596632	0.407488109	1.275238306	0.350766871

32	159.0292165	342.2565	0.522696577	0.313340698	2.295001565	0.001358973	0.165998342	1.668141358	0.738241548
33	159.0770358	303.7075	0.073226175	0.048925843	1.947634521	0.004426904	0.247142731	1.49667683	0.581762741
34	160.0400111	300.564	0.730870016	0.53174196	1.890619628	0.019245287	0.321611251	1.374482493	0.458888531
35	160.0591401	29.169	7.53502427	5.063956572	1.130335351	0.049703346	0.414421923	1.48797174	0.573347126
36	162.0562488	34.1175	9.022567249	7.21940689	1.654801368	0.018751914	0.320515166	1.24976572	0.321657674
37	163.0248107	293.8525	0.091741543	0.056785007	1.821782086	0.008047079	0.281507004	1.615594452	0.692065097
38	163.0589101	34.211	0.920115476	0.716389209	1.821801545	0.008324383	0.283110581	1.284379308	0.361071328
39	164.9776162	342.382	0.793102636	0.624493946	1.373976545	0.037296853	0.381992294	1.269992514	0.344819993
40	166.0138918	384.176	0.377712033	0.217417668	2.118932436	2.67028E-05	0.023650666	1.737264671	0.796817564
41	166.0178975	188.187	21.86984905	15.82585186	1.458003193	0.032741654	0.367881809	1.381906594	0.466660104
42	167.012945	235.534	0.941673209	0.433992252	2.318798155	0.000809581	0.13514685	2.169792673	1.117557198
43	167.0203913	187.661	0.890168977	0.663463609	1.440915286	0.043916796	0.400939185	1.341699777	0.424061886
44	167.4889032	108.628	1.466205572	0.823470326	1.445320819	0.011176741	0.295580017	1.780520226	0.832298825
45	173.008777	42.948	8.798580944	7.1193362	1.37726724	0.01162028	0.297037371	1.235870971	0.305528129
46	173.1006957	190.6355	0.610236388	0.303984064	1.954550191	0.010004728	0.291195215	2.007461771	1.005372514
47	174.4915722	42.976	0.290142762	0.224833134	1.372228685	0.013930336	0.306415756	1.290480442	0.367908277
48	175.0251691	42.951	115.7697357	95.07548789	1.207341518	0.023253563	0.334918456	1.217661232	0.284112813
49	175.0246698	300.545	1.162383458	0.862002893	1.851460514	0.04342519	0.399674788	1.348468163	0.431321461
50	176.037939	196.025	0.808135076	0.529421596	1.011893712	0.033168087	0.369054256	1.526449019	0.610179407
51	177.0400841	295.088	1.125294084	0.750818994	1.869630641	0.038349092	0.385306669	1.498755484	0.583765033
52	177.5085499	189.011	0.82594249	0.577410963	1.672829521	0.028323148	0.354226904	1.430423985	0.516442832
53	178.051274	189.011	475.588266	326.1752312	1.862074909	0.01606149	0.313533153	1.458075968	0.544065888
54	180.0555441	189.011	3.230277288	2.220560024	1.865249267	0.015198555	0.310853439	1.454712889	0.540734442
55	180.0661919	210.083	0.163603939	0.098508289	1.236659813	0.005266603	0.258318451	1.660813941	0.731890459
56	186.1130207	172.102	0.543667212	0.352638314	1.290154795	0.01899023	0.321050787	1.541713391	0.624534589
57	190.0534953	192.255	1.593298257	0.824939919	1.270894091	0.019663888	0.322503593	1.93141127	0.949655401
58	194.0467104	281.524	0.078541168	0.058023073	1.42128666	0.034899576	0.373939705	1.353619583	0.436822346
59	195.0511117	342.892	21.9443996	13.64860742	2.089610124	0.001622813	0.175602313	1.607812353	0.68509904
60	196.027939	188.5215	1.956670631	1.339810511	1.189170507	0.018193666	0.319213048	1.460408479	0.54637195
61	201.0220341	32.5715	2.908278627	1.633357559	1.323176474	0.001530415	0.172498796	1.780552342	0.832324846
62	201.0482177	24.8295	151.5499815	100.6655683	1.927836399	0.000716834	0.127559443	1.505479818	0.590223368
63	202.0265526	103.621	3.211012833	1.855366294	1.212285336	0.022784144	0.332909811	1.730662481	0.791324393
64	206.0489153	285.017	2.515688701	1.320788922	2.119066809	0.000312644	0.082059475	1.904686403	0.929553485
65	206.0817394	177.599	1.251999436	0.748655047	1.740816514	0.001494186	0.171210219	1.672331523	0.741860876

66	207.124758	304.941	0.017207091	0.011347662	1.960160919	0.007487501	0.277974695	1.51635572	0.600608233
67	212.0240398	254.717	4.260688354	2.429196286	2.071388791	0.002605843	0.207747901	1.753949806	0.810607462
68	214.9683733	288.6335	0.05644282	0.027203989	1.55685248	0.032353194	0.366793571	2.074799405	1.052971861
69	216.0875298	333.385	0.391774834	0.297557207	1.831191562	0.025009457	0.341949253	1.316637021	0.396857669
70	216.0992085	302.1025	2.257649631	1.562432205	2.079676489	0.011465115	0.296538748	1.444958459	0.531028017
71	218.0858959	282.149	0.058047062	0.042830658	1.463520427	0.006864121	0.273492804	1.355268964	0.438579195
72	221.0661207	295.361	0.158417412	0.113340244	1.562384594	0.003958819	0.239459955	1.397715461	0.483070695
73	221.0829491	173.4315	0.050123038	0.032741183	1.471519988	0.029650253	0.358642035	1.530886595	0.614367415
74	221.1023264	286.0295	0.126568338	0.083571418	1.156494189	0.019240622	0.321601116	1.514493122	0.598835026
75	222.0763467	210.662	1.38650727	0.740092463	1.643834961	0.000479521	0.102661108	1.87342439	0.905677753
76	225.1236489	415.88	0.054928139	0.011843823	2.438132553	1.00412E-05	0.015296932	4.637703341	2.213410538
77	225.1350551	304.873	0.029315818	0.019021841	1.85173741	0.023572998	0.336252847	1.541166184	0.624022436
78	226.0827787	197.5695	10.25461307	7.075764275	1.667128527	0.040945986	0.392971611	1.449258719	0.535315166
79	229.0459799	25.026	7.456625543	5.249714561	1.51265168	0.005320428	0.258942962	1.420386853	0.506283912
80	229.1298374	349.778	0.051161906	0.042742254	1.136554489	0.029025985	0.356601865	1.196986622	0.259407029
81	230.0664142	348.229	0.513163158	0.38076672	1.729721816	0.009629475	0.289598688	1.347710109	0.430510208
82	230.1756565	303.483	0.020630836	0.016052888	1.252532324	0.037015601	0.38108454	1.285179045	0.361969363
83	231.0988419	294.607	10.06376617	8.152666335	1.149789865	0.035664087	0.376587309	1.234414087	0.303826431
84	231.9925964	212.0435	81.70797155	43.09790153	1.56359724	0.001268639	0.162115184	1.895868909	0.922859211
85	236.9703809	46.833	20.26323568	8.482629336	2.288672921	0.002198072	0.193815354	2.388791833	1.256281138
86	237.0399559	294.45	0.813537425	0.582568257	1.33515471	0.038719617	0.386443974	1.396467134	0.48178162
87	239.092079	288.644	1.380075963	1.020319738	1.869707858	0.0183379	0.319556042	1.352591656	0.435726359
88	239.9980627	32.291	240.1869799	163.7408221	1.715929907	0.020088169	0.323518221	1.466872932	0.552743903
89	241.2719047	34.426	0.962943153	0.763448815	1.826300427	0.005930837	0.265404708	1.261306763	0.334919197
90	242.0137511	34.385	493.050722	409.7353741	1.634424956	0.011217572	0.295718375	1.203339407	0.267043618
91	245.1025232	39.747	0.137619207	0.207521705	1.995962824	0.017826294	0.318317838	0.663155728	-0.592580399
92	245.1387167	332.121	0.164058266	0.109744825	1.979919933	0.025246312	0.342843458	1.494906625	0.580055373
93	246.0974303	350.6815	0.208885576	0.126807019	1.30789663	0.044488622	0.402384482	1.647271404	0.720078272
94	247.0672773	424.493	0.193817914	0.107925168	1.424707925	0.021794438	0.328478215	1.795854632	0.844670574
95	247.0931649	290.344	0.205402239	0.145748608	1.748110591	0.019704001	0.322587365	1.409291254	0.494969799
96	248.0605669	338.21	0.34827382	0.211700207	2.094219003	0.020232112	0.323859238	1.645127436	0.718199344
97	249.0211522	279.7585	0.84983107	0.407167544	1.570861866	0.023882378	0.337520982	2.087177828	1.061553523
98	252.036038	197.481	1.858271014	1.206783387	1.881139158	0.004982592	0.254855255	1.539854653	0.622794181
99	252.96488	190.3545	0.372662374	0.133468885	1.642571791	0.01723577	0.316810309	2.79212923	1.481365716

100	253.0346587	295.908	0.341313802	0.224879861	1.949693051	0.018830829	0.320693831	1.517760643	0.60194429
101	253.0644361	121.373	1.518160344	0.545024955	1.780701189	0.008612093	0.284682715	2.785487766	1.47792998
102	253.1077481	274.8965	0.821803027	0.634962829	1.118961513	0.049170609	0.413279146	1.294253757	0.372120506
103	254.9345765	221.1385	0.042267114	0.031280191	1.697179952	0.005489866	0.260847427	1.351242207	0.434286298
104	254.9809189	344.906	0.249336455	0.168288141	1.758903994	0.004628655	0.250100764	1.481604429	0.567160316
105	255.7962504	42.33	1.312033324	0.965417126	1.958528031	0.005304059	0.258754061	1.359032575	0.442580037
106	255.9914074	127.122	9.115300732	7.188534016	1.613526982	0.031199564	0.363442957	1.268033331	0.342592668
107	257.0480338	267.788	0.052565548	0.028160464	1.623953946	0.012446816	0.300292419	1.866643509	0.900446428
108	257.1019538	325.337	0.133078676	0.098257781	1.792924228	0.007257762	0.276395951	1.354383077	0.437635852
109	258.9913507	190.043	11.71918293	7.083211242	1.419610548	0.013662133	0.305390085	1.654501402	0.726396514
110	259.0125997	272.9225	0.832475297	0.60959971	1.357216523	0.040344581	0.391258252	1.365609733	0.449545246
111	261.0876751	232.97	3.114402234	1.200151329	1.661605619	0.002596221	0.207449958	2.595007946	1.375738956
112	262.0907619	233.047	0.657183278	0.297321239	1.623010578	0.000393901	0.093138716	2.21034757	1.144273247
113	266.0180877	279.665	0.177229843	0.122874044	1.80208765	0.009422466	0.28867164	1.442370063	0.528441359
114	266.9808906	278.416	0.091014674	0.062474739	1.669109602	0.007504139	0.278085952	1.456823605	0.542826204
115	268.081583	325.964	0.13639592	0.094437305	1.682235059	0.044621234	0.402715831	1.444301283	0.530371722
116	268.9974203	267.455	4.190808588	2.776219388	1.756346637	0.025087357	0.342244697	1.509537973	0.594107049
117	269.0694517	321.77	0.185673369	0.13389187	1.896229087	0.008072076	0.281655308	1.386741178	0.471698547
118	269.1135429	415.8565	0.106882365	0.024386461	2.424531609	4.85247E-05	0.02855791	4.382856834	2.131871554
119	269.1133373	334.825	0.288312875	0.152232684	2.160001162	0.018727251	0.320459059	1.893896028	0.921357131
120	269.125609	305.0125	4.11474575	2.755643241	1.994332378	0.013609727	0.305185773	1.49320699	0.578414168
121	269.2109489	48.835	0.197231609	0.11662744	1.929660604	0.044113633	0.401439743	1.691125251	0.757983515
122	270.0063947	223.098	0.128258118	0.083287483	1.117358065	0.033181056	0.369089557	1.539944712	0.622878555
123	270.0101907	241.697	0.117658342	0.080475832	1.137401946	0.019000497	0.321073601	1.462033243	0.547976115
124	270.0410208	337.584	0.091828736	0.06285157	2.043880458	0.011342738	0.296137091	1.461041244	0.546996905
125	271.0297594	231.257	0.102697232	0.075853058	1.65042238	0.02537621	0.343328728	1.353897053	0.437118044
126	271.0930325	322.4045	0.588860354	0.490900535	1.042666858	0.028535752	0.354954281	1.199551256	0.262494804
127	271.129272	284.288	0.865418596	0.641895183	1.424212074	0.029080557	0.356782763	1.348224163	0.431060387
128	271.2268915	49.714	0.153742609	0.102986587	1.843571143	0.028859973	0.356048495	1.492841096	0.578060608
129	272.0575657	28.5155	0.690411177	0.539976245	1.183157422	0.014514662	0.30854081	1.278595462	0.354559879
130	272.9910972	348.2705	0.496055825	0.273100033	1.822133163	0.005648265	0.262547819	1.816388743	0.861073001
131	275.1682122	33.091	0.233457674	0.092809324	1.946794681	0.032748838	0.367901751	2.515454958	1.330819357
132	276.9623322	344.661	0.067952919	0.047846449	1.47820614	0.012000528	0.298211358	1.420229108	0.50612368
133	281.1233226	304.353	0.850661933	0.597246713	1.24694399	0.047978402	0.410653881	1.42430576	0.510258887

134	282.9908961	103.176	7.890763044	4.498346614	1.163984202	0.039828356	0.389758656	1.754147406	0.810769987
135	283.0674494	293.448	0.319416915	0.245487327	1.160851715	0.036367363	0.378955903	1.3011544	0.379792167
136	283.9866004	188.5215	15.40071909	6.859505119	1.719337309	0.000200947	0.062141235	2.24516475	1.166821313
137	285.0541217	136.374	3.947007083	2.047655983	1.408494278	0.031655323	0.364788452	1.927573341	0.946785753
138	286.0023331	249.3235	0.724768791	0.483086085	1.084661054	0.044401076	0.402164954	1.500289109	0.585240538
139	286.0589304	340.5225	0.550438586	0.317201284	1.954001645	0.003012875	0.219224225	1.735297467	0.795182993
140	286.9726719	342.475	2.5611901	2.047943955	1.401240176	0.026485513	0.347593623	1.250615327	0.322638104
141	287.0510941	292.485	0.838410674	0.530569097	1.77447252	0.027519157	0.351403147	1.580210152	0.660116435
142	287.1240234	364.013	0.223260927	0.141430257	1.413264854	0.010625024	0.293620218	1.578593797	0.658639985
143	291.1227228	280.45	0.444165601	0.273607228	1.734846509	0.008095448	0.281793283	1.623369394	0.698991319
144	293.0770567	219.839	2.244568662	1.338283103	1.369081578	0.002984219	0.218482259	1.67720018	0.74605489
145	294.9729489	344.9515	0.146234688	0.083232232	1.341552529	0.020703029	0.324946532	1.756947808	0.813071335
146	297.1181592	232.398	0.201296995	0.401870295	1.627421602	0.042249867	0.396566888	0.50090041	-0.997404304
147	297.190737	29.167	0.324295431	0.143397534	1.829485353	0.049478395	0.413941613	2.261513304	1.177288483
148	302.0538761	236.428	0.661127847	0.451339024	1.859015998	0.003905786	0.238507838	1.464814281	0.550717761
149	303.1293946	29.771	0.745370377	0.405720692	1.961907433	0.010014932	0.291237192	1.837151499	0.877470602
150	305.0437384	418.8425	0.156820313	0.07874769	1.339819079	0.0146897	0.309149858	1.991427465	0.993802933
151	307.030202	37.111	0.767132697	0.544734999	1.638196707	0.008905397	0.286197187	1.408267687	0.493921591
152	310.0962618	325.817	4.203339463	2.835717141	1.619733168	0.003343623	0.227171531	1.482284464	0.567822341
153	311.208576	28.5155	1.007711549	0.456164381	1.67724663	0.049484758	0.413955244	2.209097403	1.143457031
154	311.9807897	252.003	0.022924215	0.012029843	1.920815212	0.002283265	0.196964998	1.905612146	0.930254514
155	312.0004335	340.873	0.366576637	0.230463602	1.761057898	0.003908479	0.238556634	1.590605341	0.66957592
156	312.0931919	325.433	0.15989412	0.118278512	1.616664566	0.014245553	0.307580143	1.351844194	0.434928885
157	312.9860385	411.0105	0.036838385	0.023298166	1.0299158	0.027371	0.350869794	1.581171003	0.660993403
158	315.0131045	275.736	0.256829103	0.11670236	1.946219018	0.003465559	0.229842101	2.200719009	1.137974952
159	315.0129968	196.071	1.862888996	0.497153063	2.419049259	0.014782468	0.309467751	3.747113582	1.905779709
160	323.0062464	350.482	0.067884687	0.050700832	1.794902486	0.005644317	0.262506334	1.338926481	0.421076746
161	323.1337307	287.036	0.24057771	0.178479935	1.59312704	0.017931519	0.318577481	1.347925809	0.430741092
162	325.0362995	318.458	0.12805771	0.08713888	1.894552505	0.014429684	0.308240682	1.46958178	0.555405645
163	325.2258838	28.033	1.248331997	0.526012049	1.846483377	0.033616972	0.370264084	2.373200387	1.246833923
164	326.0909736	361.5935	0.83826168	0.598464249	1.155998134	0.018772953	0.320562925	1.40068798	0.486135614
165	329.086415	285.9695	0.413006135	0.279303341	1.315339702	0.040963787	0.393021785	1.478701018	0.56433038
166	330.1293788	356.2765	0.095039839	0.075978708	1.452707304	0.025411214	0.34345888	1.250874639	0.322937212
167	331.0114798	279.745	0.08043767	0.04862119	1.830997703	0.004060656	0.241238661	1.654374791	0.726286107

168	334.9370837	42.935	5.849790584	4.46585614	1.067327291	0.04648244	0.40722032	1.309892303	0.389448201
169	335.9639208	212.2945	0.148374911	0.107420786	1.365960603	0.014922107	0.309940013	1.381249538	0.465973982
170	336.0722864	300.253	15.35010509	10.82640146	1.877056196	0.030399533	0.361009007	1.417840004	0.503694741
171	339.2432232	27.489	0.785459571	0.321039564	1.78784012	0.03373297	0.370572748	2.446612999	1.290785916
172	342.0818137	292.4645	0.658514349	0.539915421	1.492849082	0.039029525	0.387383678	1.21966205	0.286481453
173	343.1019705	276.9875	0.62232631	0.421556754	1.288984054	0.019583882	0.322335614	1.476257476	0.561944365
174	343.1107912	260.188	0.16173819	0.106352338	2.151310325	0.009645826	0.289670464	1.520777001	0.604808619
175	343.159981	313.397	0.079983617	0.036613724	2.088700134	0.002007441	0.186235883	2.18452559	1.127320006
176	343.1603654	350.489	0.053055633	0.039076936	1.445060442	0.020311381	0.324045272	1.357722455	0.441188595
177	344.0384369	301.961	0.074102659	0.049856952	2.054167449	0.016308361	0.314255423	1.486305435	0.57173062
178	349.057105	350.539	0.479984218	0.342420327	2.022694932	0.011450924	0.296492557	1.401739849	0.487218623
179	349.1305675	333.117	0.083697324	0.054143197	2.223145206	0.014975025	0.310117051	1.545851146	0.628401405
180	350.1083891	288.482	3.496883988	2.45923117	1.706708066	0.023527933	0.33606615	1.421941959	0.507862578
181	355.0657406	359.392	0.109673801	0.077467603	1.288107904	0.021210846	0.326072567	1.415737629	0.501553923
182	355.1202529	139.5115	0.228726719	0.147323519	1.504696953	0.025470311	0.343678022	1.55254721	0.634637139
183	358.1602192	326.3195	0.167504729	0.12864575	1.389372576	0.020766431	0.325089697	1.3020619	0.380798036
184	362.1245653	260.6015	0.265397956	0.217844227	1.375200979	0.041093268	0.393385804	1.218292355	0.28486038
185	363.0235081	128.2175	3.219174992	6.171334807	1.96245774	0.03130869	0.363767775	0.521633503	-0.938891563
186	365.017465	296.57	0.070193764	0.04877281	1.621147239	0.02881058	0.355882956	1.439198692	0.52526578
187	365.0170816	279.546	0.667322944	0.471092827	1.359934053	0.029151245	0.357016348	1.416542357	0.502373741
188	365.068176	117.2255	9.665908936	5.598789182	1.55763598	0.005655201	0.262620589	1.726428451	0.787790546
189	373.0339995	252.3215	1.411975581	0.676997024	1.736452423	0.001359419	0.16601667	2.085645182	1.060493742
190	374.0347782	279.6555	0.339309113	0.160176456	1.506953111	0.025595484	0.344139764	2.118345734	1.08293807
191	375.0490803	238.551	0.488146787	0.305840491	1.34278303	0.004490056	0.248089564	1.596082933	0.674535616
192	375.150388	293.114	0.243949446	0.151287528	1.865410468	0.011736873	0.297404432	1.612488805	0.689289144
193	379.9944905	275.725	0.38921865	0.267581981	1.668897637	0.006368064	0.269428309	1.454577206	0.540599874
194	380.0616482	361.5935	0.292381811	0.178137865	1.289000277	0.029267598	0.357399033	1.641323206	0.714859359
195	381.1497824	260.557	0.809618444	0.622253989	1.159208279	0.012908055	0.302319526	1.301106073	0.379738583
196	386.1186816	307.8005	0.147243559	0.107806471	1.736585181	0.018181267	0.319183345	1.365813745	0.449760758
197	388.126812	284.674	0.210331896	0.118393491	1.461002644	0.006960646	0.274229258	1.776549501	0.829077887
198	388.1704665	329.533	0.038138838	0.027614981	1.528210736	0.015165298	0.310745037	1.381092308	0.465809748
199	389.1758189	329.632	0.015775477	0.012230207	1.175858105	0.024419388	0.339667449	1.289878202	0.367234844
200	393.0632631	126.767	0.557039139	0.355437276	2.072547414	0.030153624	0.360241743	1.567193926	0.648183711
201	394.066153	370.1615	0.041449691	0.029334869	1.240866869	0.038281315	0.385096982	1.412983685	0.498744808

202	396.0040899	279.953	0.190076647	0.13282723	1.803507846	0.013585249	0.305089895	1.431006629	0.517030355
203	397.0792546	370.4335	0.509847617	0.35737279	1.240340757	0.036788294	0.380343963	1.426654829	0.512636325
204	397.1122069	173.601	0.318922091	0.213524103	1.749896955	0.018243497	0.319332077	1.493611667	0.578805102
205	398.1578919	281.591	0.06163709	0.038373646	1.25471433	0.010436364	0.292909009	1.60623493	0.683682919
206	400.0627636	290.41	0.121903607	0.058521868	1.985065044	0.000463511	0.101019933	2.083043692	1.0586931
207	403.1058923	24.369	0.418782101	0.193761509	1.967912809	7.25976E-05	0.031183241	2.161327621	1.111917777
208	407.0533312	32.616	0.292038953	0.198925793	1.347784995	0.016253245	0.314095786	1.468079871	0.553930461
209	410.0852724	157.243	0.385915391	0.273775755	1.971542492	0.014118671	0.307116645	1.409603967	0.495289889
210	411.1073774	299.425	0.442359338	0.289155412	1.757751917	0.001365439	0.166263208	1.52983247	0.613373674
211	413.1910849	102.0655	2.434155447	1.310823153	1.666534956	0.048386022	0.411562234	1.856967084	0.892948242
212	418.1005657	366.6325	0.096118161	0.038797934	1.469175299	0.027522674	0.351415756	2.477404126	1.308829227
213	419.0005067	343.418	0.014300423	0.011014865	1.188078173	0.009221182	0.287736373	1.298284035	0.376606046
214	420.2039368	113.86	0.252806405	0.507901043	1.717309529	0.017148043	0.316578769	0.497747364	-1.00651442
215	423.2361793	51.532	0.43540157	0.210457611	2.168946555	0.001757538	0.179707442	2.068832613	1.048816923
216	425.203071	303.407	0.021844332	0.016031001	1.617570943	0.019213136	0.321541315	1.362630523	0.446394428
217	433.1919339	293.919	0.084711946	0.055545062	1.646580807	0.009640222	0.289645889	1.525103109	0.608906784
218	437.1396265	333.033	0.089842194	0.063759551	1.867051077	0.025283026	0.342980979	1.409078224	0.494751704
219	442.1202515	258.047	0.370825204	0.749724758	2.033398387	0.034429304	0.372392343	0.494615124	-1.01562174
220	447.1340881	328.8495	0.12391661	0.085606812	1.819491387	0.015817556	0.312800737	1.447508746	0.533572066
221	448.1110201	396.282	0.225276699	0.109812061	1.086065849	0.036784253	0.380330739	2.051475003	1.036661575
222	453.0661166	28.816	1.565383539	1.171863307	1.368924275	0.030817604	0.362292601	1.335807282	0.417711884
223	461.2343401	292.0785	0.118767995	0.059136479	1.868364749	0.00450681	0.248337498	2.008371074	1.006025851
224	464.0246049	82.197	1.465972019	0.533647638	2.5570252	7.97556E-05	0.031710629	2.747078623	1.457898202
225	464.9900219	211.998	0.051910239	0.017307084	1.237138436	0.006978046	0.274360262	2.999363653	1.58465645
226	465.0747075	234.8955	0.177469588	0.134637845	1.522770448	0.009556314	0.289274966	1.318125587	0.398487832
227	475.0640698	234.2045	0.300705997	0.206963164	1.676994047	0.02346431	0.33580171	1.452944532	0.538979627
228	477.0874591	246.025	0.041422549	0.020608141	1.039557167	0.034548429	0.372698026	2.010009008	1.007201967
229	477.2178076	294.6205	0.083536268	0.053529295	1.639674762	0.034538664	0.372673028	1.560571059	0.642074051
230	483.2176038	302.659	0.044675369	0.029633305	1.523090518	0.008452735	0.283823	1.507606682	0.592260095
231	484.9799489	218.011	0.026683254	0.014625763	1.718703147	0.011425445	0.296409371	1.824400783	0.867422694
232	485.1862104	333.404	0.026993134	0.019073661	1.878585621	0.019052311	0.321188405	1.415204674	0.501010718
233	486.9707237	212.07	0.213622742	0.106787997	1.495012712	7.44116E-05	0.03132475	2.000437761	1.000315743
234	491.1595019	329.791	0.079273779	0.061410838	1.640662689	0.033145517	0.368992773	1.290876022	0.368350449
235	494.9530543	453.596	0.126702441	0.082675919	1.535854343	0.031831324	0.365300335	1.532519286	0.61590523

236	499.0495183	279.835	0.672990115	0.306838264	1.557744224	0.027622032	0.351771065	2.193305703	1.133106909
237	503.0589262	234.6285	0.187278561	0.115938093	1.75746536	0.009908441	0.290795446	1.615332429	0.691831096
238	506.829555	343.298	0.152153138	0.106024944	1.832515651	0.037305119	0.38201883	1.435069264	0.521120371
239	507.0135252	34.571	0.406313884	0.341903389	1.698040506	0.044825837	0.403224273	1.188387999	0.249005941
240	510.0636528	300.792	0.069926721	0.026748523	2.005183489	0.007486806	0.27797004	2.614227378	1.386384628
241	510.9685492	42.6445	1.732643568	1.223309851	1.56093379	0.011437446	0.296448594	1.416357079	0.50218503
242	512.1512744	288.689	0.049086702	0.037142017	1.44129409	0.043651365	0.400259049	1.321594933	0.40228006
243	513.012302	111.056	0.170003144	0.10948844	1.519454534	0.024917207	0.341597662	1.552704048	0.634782872
244	521.0289571	279.786	0.041704573	0.030631732	1.380744728	0.019206565	0.321526996	1.361482702	0.445178652
245	527.9949047	42.3575	1.623355905	1.117842071	1.66855136	0.009549295	0.289243684	1.452222945	0.538262952
246	530.0891286	34.7825	0.27024078	0.220520377	1.449357878	0.020042344	0.323408782	1.225468517	0.293333421
247	532.1832867	328.377	0.047812131	0.032926458	1.300073734	0.021030499	0.325678006	1.452088502	0.538129386
248	537.3417506	103.894	0.164262575	0.080826329	1.692084593	0.01581491	0.312792688	2.032290425	1.023106586
249	550.1404348	197.574	0.283021789	0.158970212	1.371156847	0.02182889	0.328637162	1.780344794	0.832156671
250	551.3211881	103.858	0.127343672	0.050797488	2.084126506	0.004524358	0.24859573	2.506889162	1.325898211
251	553.3361814	107.056	0.084287941	0.045809703	2.000207414	0.001158982	0.156891235	1.839958244	0.879673026
252	554.033521	196.081	0.011389024	0.022654694	1.397581059	0.034318675	0.372107015	0.502722471	-0.992165918
253	560.9752461	241.0205	0.056646999	0.036050223	1.500125543	0.025175912	0.342578947	1.57133559	0.65199133
254	567.1050879	457.1535	0.028025412	0.01996071	1.15507971	0.011337529	0.296119828	1.404028816	0.489572546
255	572.0711813	275.625	0.02493958	0.011665922	1.660451912	0.021266563	0.326193302	2.137814834	1.0961369
256	575.1173718	190.271	0.072338654	0.04154877	1.576795168	0.037835564	0.383705023	1.741054054	0.799960994
257	586.1273429	374.639	0.02131513	0.011524028	1.231265947	0.017036069	0.316280267	1.849624872	0.887232703
258	588.1309392	81.531	0.127828677	0.268745449	2.089829375	0.017955147	0.318635421	0.475649643	-1.072028801
259	590.9256568	42.9495	0.159260482	0.122316784	1.114216772	0.035515755	0.376079638	1.302032945	0.380765953
260	603.0326414	221.889	0.013090634	0.007017366	1.698401036	0.011052077	0.295152077	1.865462672	0.899533492
261	607.9525452	42.3575	0.205289775	0.138826288	1.393921363	0.024691022	0.340727607	1.47875289	0.564380988
262	617.182419	196.9015	0.306034585	0.20186013	1.883366616	0.031881773	0.365446283	1.51607247	0.600338717
263	624.0647254	279.835	0.246199159	0.11212474	1.551560322	0.024941615	0.341690871	2.195761236	1.134721186
264	636.0862562	53.4045	0.18441555	0.126041881	1.440703791	0.023983361	0.337929855	1.463129146	0.549057118
265	641.2524484	349.8795	0.014393552	0.011007526	2.047453025	0.001766367	0.179960757	1.30761012	0.386932448
266	647.1348601	97.256	0.322478053	0.190141625	1.085408606	0.013528643	0.304867083	1.69598873	0.762126583
267	649.1770815	160.69	0.344351934	0.242058836	1.594684248	0.018128259	0.319055956	1.422596007	0.508526019
268	657.2205558	139.082	0.176939143	0.083567337	1.329571395	0.039300536	0.38819696	2.117324183	1.082242177
269	687.1772566	96.135	2.052743832	1.557092427	1.181539845	0.049762215	0.414547085	1.318318551	0.398699017

270	706.0863641	217.985	0.007629533	0.004667456	1.298462418	0.033904344	0.371025832	1.634623351	0.708958249
271	707.2293923	203.133	0.03923139	0.027210029	1.762648966	0.01865019	0.320282925	1.441798888	0.527869942
272	721.1650138	95.17	0.394373229	0.25493373	2.213262281	0.004089176	0.241725472	1.546963712	0.629439355
273	725.1340573	95.837	0.527106474	0.401796322	1.848235139	0.011856072	0.297773149	1.311874811	0.391630054
274	728.0524226	34.356	0.088020637	0.056816176	1.201639066	0.0289298	0.356281815	1.549217912	0.631540087
275	735.1801922	93.51	0.3432498	0.207361841	1.597928269	0.009125857	0.287281253	1.655318057	0.727108447
276	739.1501096	94.6795	0.717852021	0.551481554	1.746303872	0.02100448	0.325620602	1.301679115	0.380373845
277	739.2077107	427.284	0.108531085	0.042286974	1.353861817	0.007605576	0.278755594	2.566537021	1.359823071
278	749.0799759	279.745	0.256042339	0.109928239	1.466906662	0.028336608	0.354273187	2.329177114	1.219820349
279	761.1884082	427.503	0.0099396	0.005276731	1.188213993	0.012478372	0.300434985	1.883666297	0.913543405
280	771.0534879	279.758	0.044450701	0.029190527	1.583473238	0.011323756	0.29607411	1.522778296	0.606705913
281	773.1098923	34.0335	0.026934234	0.019290668	1.796665402	0.021964969	0.329261597	1.396231276	0.481537934
282	801.0111668	42.894	0.528761995	0.341273868	1.64390768	0.015072705	0.310441102	1.549377331	0.631688537
283	819.1640571	430.429	0.017069822	0.007465767	1.46026597	0.016675841	0.315296773	2.286412434	1.193085667
284	874.094159	279.953	0.207428571	0.088755304	1.486385125	0.035418318	0.375744597	2.337083659	1.224709378
285	896.0727934	279.835	0.019667749	0.013689925	1.455274852	0.017334057	0.317067334	1.436658571	0.522717239
286	898.1549436	439.635	0.020644053	0.010138629	1.301767953	0.045798739	0.405596638	2.036177954	1.025863653
287	904.1049206	279.4475	0.007919704	0.005023577	1.213290599	0.04510734	0.403918339	1.576507139	0.656731703
288	904.5912297	312.0475	0.047369732	0.035024119	1.298482157	0.010302778	0.292391837	1.352488886	0.435616739
289	915.2757767	196.859	0.216552909	0.137162798	1.844613328	0.046553932	0.407388087	1.578802062	0.658830309
290	930.0670673	409.3265	0.010527322	0.006249524	1.548997376	0.017824179	0.318312592	1.684499874	0.75232032
291	999.1118062	279.884	0.098759531	0.042737953	1.394869288	0.044977595	0.403599226	2.31081565	1.208402171
292	1021.086528	279.953	0.020255872	0.014144697	1.345400326	0.04575213	0.405484663	1.432047088	0.518078932
293	1023.083053	279.745	0.012263485	0.007377909	1.383510363	0.012633186	0.301126016	1.662189921	0.733085233
294	1061.621772	279.741	0.011894046	0.002494161	1.483205444	0.038670061	0.386292742	4.768755877	2.25361293
295	1074.026387	42.3575	0.206480999	0.12190848	1.84189538	0.011890234	0.297877623	1.693737781	0.760210539
296	1124.125842	279.645	0.060088829	0.026516896	1.593513521	0.037999592	0.38421986	2.266058159	1.180184889
297	1146.100397	279.771	0.014833451	0.008765896	1.493670993	0.005055725	0.25577507	1.692177446	0.758880861
298	1148.093034	279.9575	0.008401901	0.005167767	1.357061335	0.006494163	0.270508455	1.625827893	0.701174545

Note: The metabolites were selected by the condition of VIP > 1. $p < 0.05$.
